# Do Invasive Earthworms Affect the Functional Traits of Native Plants?

**DOI:** 10.3389/fpls.2021.627573

**Published:** 2021-03-16

**Authors:** Lise Thouvenot, Olga Ferlian, Rémy Beugnon, Tom Künne, Alfred Lochner, Madhav P. Thakur, Manfred Türke, Nico Eisenhauer

**Affiliations:** ^1^German Centre for Integrative Biodiversity Research (iDiv) Halle-Jena-Leipzig, Leipzig, Germany; ^2^Institute of Biology, Leipzig University, Leipzig, Germany; ^3^Terrestrial Ecology Group, Institute of Ecology and Evolution, University of Bern, Bern, Switzerland

**Keywords:** biological invasion, biotic interactions, exotic earthworms, iDiv Ecotron, plant functional traits, plant–soil interactions

## Abstract

As ecosystem engineers, invasive earthworms are one of the main drivers of plant community changes in North American forests previously devoid of earthworms. One explanation for these community changes is the effects of earthworms on the reproduction, recruitment, and development of plant species. However, few studies have investigated functional trait responses of native plants to earthworm invasion to explain the mechanisms underlying community changes. In a mesocosm (Ecotron) experiment, we set up a plant community composed of two herb and two grass species commonly found in northern North American forests under two earthworm treatments (presence vs. absence). We measured earthworm effects on above- and belowground plant biomass and functional traits after 3 months of experiment. Our results showed that earthworm presence did not significantly affect plant community biomass and cover. Furthermore, only four out of the fifteen above- and belowground traits measured were affected by earthworm presence. While some traits, such as the production of ramets, the carbon and nitrogen content of leaves, responded similarly between and within functional groups in the presence or absence of earthworms, we observed opposite responses for other traits, such as height, specific leaf area, and root length within some functional groups in the presence of earthworms. Plant trait responses were thus species-specific, although the two grass species showed a more pronounced response to earthworm presence with changes in their leaf traits than herb species. Overall, earthworms affected some functional traits related to resource uptake abilities of plants and thus could change plant competition outcomes over time, which could be an explanation of plant community changes observed in invaded ecosystems.

## Introduction

Biological invasions are one of the most important threats to native biodiversity (Davis, 2009; Wardle et al., 2011; Díaz et al., 2019). Among them, invasive ecosystem engineers, such as earthworms, can be particularly harmful (Bohlen et al., 2004; Frelich et al., 2019). They affect soil properties (Ferlian et al., 2020), native soil fauna (Eisenhauer, 2010; Ferlian et al., 2018), and plant biodiversity (Craven et al., 2017), with consequences for other trophic levels and ecosystem services (Frelich et al., 2012, 2019). These effects are even stronger when recipient ecosystems lack functionally similar native soil fauna (Wardle et al., 2011), such as the case for earthworms in many regions in northern North America. There, the native earthworm fauna is assumed to have disappeared during the last glaciation, prior to the recent introduction of exotic earthworm species ∼400 years ago (Bohlen et al., 2004; James and Hendrix, 2004; Hendrix et al., 2008).

A large portion of previous research on the ecosystem consequences of earthworm invasion in northern North America has focused on plant community composition and diversity (Hale et al., 2006; Nuzzo et al., 2009, 2015; Hopfensperger et al., 2011; Drouin et al., 2016; Craven et al., 2017). The direct and indirect mechanisms behind observed plant community changes are manifold. Invasive earthworms influence seed bank composition, seed recruitment, and the development and survival of seedlings (Hopfensperger et al., 2011; Clause et al., 2015; Dobson and Blossey, 2015; Nuzzo et al., 2015; Cassin and Kotanen, 2016). The outcome of these earthworm–seed interactions may depend on the morphological and chemical traits of seeds (Eisenhauer et al., 2009c; Clause et al., 2011, 2017). Moreover, invasive earthworms can further alter the plant functional traits of native plants. Exotic earthworms induce changes in the above- and belowground biomass (Hale et al., 2008), stem height, number of leaves and culms (Dávalos et al., 2013, 2015), nutrient concentrations of tissues (Dobson et al., 2017; Richardson et al., 2018), as well as myccorhizae association (Paudel et al., 2016). However, plant responses depend on the identity of the plant species and their functional groups (Kreuzer et al., 2004; Eisenhauer and Scheu, 2008; Coulis et al., 2014), and may further vary according to the species’ phenotypic plasticity. Currently, we lack information about potential changes of a set of plant functional traits to better understand plant responses to exotic earthworm presence, and consequently the mechanisms behind native plant community changes.

Earthworms are likely to alter soil nutrient availability and dynamics, such as changing the spatial distribution of nutrients from homogeneous to patchy (Shuster et al., 2001) or the distribution between soil layers (Ferlian et al., 2020). Besides, invasive earthworms were shown to increase the drainage/leaching of soil nutrients in their burrows (Larson et al., 2010; Eisenhauer et al., 2012a). Thus, we could expect that plant root traits respond to these earthworm-induced soil modifications, and that the changes in native plant community composition due to earthworm invasion are related to their selective effects on particular nutrient resource uptake strategies of plants (Andriuzzi et al., 2016).

Indeed, some plant species, such as the native species *Achillea millefolium*, were shown to place their roots inside the burrows of exotic earthworms (Cameron et al., 2014). As a consequence, exotic earthworms may specifically foster plant species with the highest ability to take up resources in their burrows and that have the highest plasticity in their functional traits. Such plant species are likely to be located toward the ‘fast end’ of the whole-plant economic spectrum (Reich, 2014). Fast-growing species are characterized by, e.g., a low leaf dry matter content, high specific leaf area and nitrogen content in leaves (Craine, 2009; Donovan et al., 2011; Reich, 2014), as well as a high specific root length and percentage of fine roots (Tjoelker et al., 2005; Roumet et al., 2016), with these characteristics being linked to high nutrient uptake and assimilation abilities. Consequently, earthworm-induced habitat changes could favor species with high adaptation and resource competition abilities under high resource conditions, such as fast-growing species like nitrophilous graminoid species (Craven et al., 2017).

Here, using a plant functional trait approach and plant species originating from the understory community of a North American boreal forest, we studied plant species responses to exotic earthworms to advance our understanding of the mechanisms behind native plant community changes due to earthworm invasion. We hypothesize that (i) earthworms will increase plant community biomass and cover (mostly driven by a positive response of grasses) by increasing soil nutrient availability; (ii) earthworms will mainly affect the traits related to resource uptake (e.g., height, specific leaf area, and specific root length); and (iii) plant functional trait responses will vary according to plant species identity but will be more pronounced in grass species (i.e., fast-growing species) than in herb species.

## Materials and Methods

### Experimental Design

We investigated how exotic earthworms affect plant functional traits in an indoor mesocosm experiment. We simulated the conditions of a well-studied Canadian aspen forest that is known to experience earthworm invasion and subsequent shifts in plant communities (Eisenhauer et al., 2007, 2009d; Straube et al., 2009). The experiment was conducted at the iDiv Ecotron of the German Centre for Integrative Biodiversity Research (iDiv) Halle-Jena-Leipzig in Germany (Eisenhauer and Türke, 2018). We used twelve EcoUnits (i.e., experimental units) set up in an air temperature-controlled environment. For each of the EcoUnits, the air temperature was on average 18.5 ± 2.3°C at day (± sd) and 15.7 ± 1.7°C at night (mean ± sd), in a 16 h day/8 h night cycle, with a light intensity of around 400 μmol m^–2^ s^–1^ of photosynthetic photon flux density at the soil surface. The soil temperature was on average 17.1 ± 1°C. The EcoUnits were irrigated using a sprinkler system delivering 1 L of tap water in ∼1 min, every 6 h. The amount of tap water used was adjusted regularly and simultaneously for each EcoUnit and ranged between 0.5 L to 1 L in order to keep the volumetric soil water content lower than 40%.

The bottom part of each EcoUnit (inner dimension (L × W × H): 1.24 × 1.24 × 0.8 m) was filled with 1.23 m^3^ of sterilized topsoil (pH = 7.05; C:N = 9.30) provided by Bauzentrum Farys GmbH, Laucha. Prior to filling the EcoUnits, the soil was sterilized by steam heating (120°C for 1 h) and was then heavily watered to drain the nutrients resulting from the sterilization procedure. Afterward, the soil was re-inoculated with a microbial wash of the soil from the earthworm-free area of the above-mentioned aspen forest in Kananaskis Valley (Alberta, Canada) to introduce soil microbial communities without any co-evolutionary history with European earthworms. To do so, Canadian soil was collected in the field, shipped to Germany in a cooling box, sieved (4 mm mesh size), and homogenized. We then mixed 380 g of this soil with 300 mL of tap water and homogenized and sieved (125 μm mesh size) the solution. The extracted solution was re-diluted with 100 ml of tap water and then distributed homogeneously on the topsoil of each EcoUnit.

The aboveground community consisted of four Canadian native plant species (grasses and herbs) and two native tree species. The seeds of grasses [*Calamagrostis canadensis* (Michx.) and *Bromus ciliatus* (L.)] were purchased from Prairie Moon Nursery (MN, United States) and the herbs [*Symphyotrichum laeve* (L.) and *A. millefolium* (L.)] from the nursery Wild About Flowers (AB, Canada). While native populations of *A. millefolium* occur in North America, exotic populations originating from Europe have also established, leading to a possible hybridization and thus difficulty to distinguish them morphologically (Mulligan and Bassett, 1959; Warwick and Black, 1982). Therefore, the reader should note that we cannot be sure to have sown native seeds of *A. millefolium* in the present study. The tree species *Populus balsamifera* (L.) and *Populus tremuloides* (Michx.) came from the tree nursery Pflanzenhandel Winkler Gbr (Germany). After careful washing of the roots with tap water to avoid the introduction of soil fauna into the EcoUnits, two individuals of each of the two tree species were planted diagonally at a distance of around 85 cm in each of the EcoUnits, adding up to four tree individuals in each EcoUnit, with one tree individual in the center of each quarter. Dead trees were replaced during the first 5 weeks and were harvested after 16 weeks of the experiment. Although the presence of trees was important to simulate the conditions in the target forest, tree responses to earthworms were not investigated in the present study, whereas we used the dry weight of the tree biomass produced per EcoUnit (dried at 60°C for 72 h, and including the biomass of trees re-sproutings) as a covariate in our models to explain plant trait- and community responses. The tree performance assessments had a different focus than the study of herbaceous plant traits (Thakur et al., 2020).

The herbaceous plant community was planted approximately 1 month after the last tree replacement to avoid disturbance due to the replacement of dead trees. The seedlings constituting the herbaceous community were planted according to two random patterns of the plant community mixture. In both patterns, the plant community consisted of 50% grasses and 50% herbs, with herbs and grasses planted in an alternate order (Supplementary Material 1). These patterns were set to introduce heterogeneity in the location of the different plant species of the plant understory community. Species within functional groups were randomly distributed to functional group locations in each pattern, keeping the number of individuals per species and EcoUnit constant (36 individuals per species, thus 144 individuals per community in one EcoUnit). The plant seedlings were pre-grown in a greenhouse for about 16–28 days at 21°C at day and 15°C at night, and were transplanted at 10 cm distances into each EcoUnit forming the experimental plant communities. The average height (± sd) of transplanted seedlings depended on the plant growth forms. Overall seedlings of herb species such as *S. laeve* that form a basal rosette (1.6 ± 0.6 cm) were smaller than that of grass species such as *B. ciliatus* that grow up erected/upright (15.5 ± 3.3 cm). *Achillea millefolium* (8.2 ± 3.5 cm) and *C. canadensis* (12.4 ± 3.7 cm) had intermediate height. Dead seedlings were replaced during the first 3 weeks, and weeds originating from the seed bank were removed regularly. None of the experimental treatments influenced the survival of the planted individuals (Supplementary Material 2).

The earthworm treatment was applied 16 days after planting the trees. Earthworms were added to half of the EcoUnits (*n* = 6 without earthworms and *n* = 6 with earthworms) and corresponded to a mixture of two ecological groups of earthworms to simulate the earthworm community and functional composition of northern North American ecosystems (Straube et al., 2009; Eisenhauer et al., 2011). To the six EcoUnits with earthworm presence, we added earthworm species from the Lumbricidae family, invasive in North America with a density of around 43 ind.m^–2^: 50 individuals of *Lumbricus terrestris* (L.) (anecic; total average fresh weight ± sd: 167.31 ± 0.92 g) and 15 individuals of *Aporrectodea rosea* (Savigny) (endogeic; 4.02 ± 0.10 g) were introduced. Earthworms were purchased from commercial suppliers (TZ – Terraristik Zentrum, Germany) or collected in a garden in the vicinity of Leipzig (Germany) and kept in the fridge (12°C) until use. To control for the potential effect of soil in earthworm guts (including nutrients and microorganisms), 4 g of soil previously used for earthworm storage was added to each EcoUnit of the control treatment without earthworms. During the experiment, we added litter on the top of the soil to provide food to earthworms and simulate natural conditions. Three times, 50 g of litter collected from *Populus nigra var. italica* stands in the vicinity of Leipzig (Germany) were added to each EcoUnit. The litter material was cut into small pieces of about 2 cm in diameter and homogeneously distributed on the soil surface. The last litter addition (this time a total of 100 g per EcoUnit) was done 40 days before the end of the experiment and consisted of a mixture of ^15^N labeled and non-labeled poplar litter. The mixture contained 5% of labeled material (4.2 ± 3.8 atom% ^15^N) and was used to explore potential earthworm-induced changes in nutrient mineralization and uptake by plants based on ^15^N signatures in plant leaves.

At the end of the experiment, we verified that earthworms successfully established and were active in our EcoUnits. Across the replicates of the earthworm treatment, we counted on average 35 ± 2.61 casts per m^2^ (mean ± sd) produced by the earthworms at the surface of the soil. We then assessed earthworm abundances by performing earthworm extraction in two opposite quarters of each EcoUnit, using the hot mustard method (Eisenhauer et al., 2008). The mustard solution was prepared by diluting 100 g of mustard powder in 10 L of tap water. In each quarter of the EcoUnit, 5 l of the mustard solution was applied to a circle of 50 cm in diameter. Earthworms appearing at the soil surface were collected during 15 min. Then, the procedure was repeated by adding another 5 l of mustard solution, and earthworms were collected for another 15 min. Then, they were sorted, counted, and weighed according to ecological groups. We extracted almost half (∼42%) of the earthworms that were initially inoculated. Across the EcoUnits where earthworms were added, the density of earthworms extracted was around 18.3 ± 4.9 ind.m^2^ (mean ± sd), and their total biomass was around 50.1 ± 9.9 g.m^2^ (mean ± sd). However, the density of earthworms in the mesocosms is probably underestimated, as the mustard extraction method is not comprehensive and also less efficient for endogeic species (Chan and Munro, 2001; Lawrence and Bowers, 2002; Gutiérrez-López et al., 2016). Indeed, extracted earthworms mainly belonged to the anecic species *L. terrestris* (17.8 ± 4.8 ind.m^2^; 50 ± 9.7 g.m^2^), while few were from the endogeic species *A. rosea* (0.4 ± 1.0 ind.m^2^; 0.1 ± 0.3 g.m^2^). These results thus indicate the successful establishment of the earthworm treatment, but does not allow any conclusions about the percentage of surviving earthworms. No earthworms were found in the control treatment.

### Data Collection

#### Plant Cover Estimation

Plant above- and belowground traits related to plant development, resource-use strategy, and reproduction ability were measured after 103 days of the experiment. Prior to trait measurements, total cover as well as the species-specific plant cover were estimated using a modified decimal scale from Londo (1976). To do so and to avoid sampling bias, two persons estimated visually and independently the cover of each community and agreed on a cover category. The same procedure was applied for the cover of each species in each mesocosm. Trees re-sproutings were included in the total cover estimation. Thirteen cover categories were defined: <1%; 1–3%; 3–5%; 5–15%; 15–25%; 25–35%; 35–45%; 45–55%; 55–65%; 65–75%; 75–85%; 85–95%; and >95%. The median values of these classes (0.5, 2, 4, 10, 20, 30, 40, 50, 60, 70, 80, 90, 98%) were used for further analyses.

#### Aboveground Traits and Species-Specific Biomass

To record the vegetative height (cm), ten individuals per species were randomly selected for each plant pattern, in the center of the EcoUnit (i.e., to avoid potential border effects) using an R function. Vegetative height was measured as the shortest distance between the soil surface and the upper part of the highest leaf of the plant (Cornelissen et al., 2003; Pérez-Harguindeguy et al., 2016). We also recorded the presence-absence of flowers as well as the number of ramets for each of these ten individuals. The mean values of these two traits were calculated per species at the EcoUnit level prior to further analysis. Among these ten individuals per species, five individuals were randomly selected in full light conditions to measure individual biomass and leaf traits. The species-specific biomasses per EcoUnit were measured by cutting shoots at 1 cm above the soil surface. Plant species-specific biomasses were dried at 60°C for at least 72 h and weighed. To calculate the total biomass of the plant community, we summed the dry weight of the four plant species, including the dry biomass of the individuals sampled for trait measurements.

Leaf traits, such as specific leaf area (SLA) and leaf dry matter content (LDMC), were measured on five leaves per individual (i.e., on the five individuals per species per EcoUnit). Plant individuals were cut at the soil surface, wrapped in moist paper, and stored in sealed plastic bags in the dark in a fridge (4°C) before being processed on the same day. Leaves that were sampled from the top to bottom of the plant, were then swabbed using paper towels to remove any surface water, and weighed (fresh mass; g), before being scanned with an Epson Perfection 11000XL Scanner (Epson America, Inc., CA, United States) at 600 dpi in grayscale. Leaves and the other biomass of individuals were then oven-dried at 60°C for at least 72 h and reweighed. Leaf areas were calculated using WinFOLIA software (Version: 2014a Pro; Regent Instruments Inc., Canada). SLA that is related to the relative growth rate of the plant and its investment into structural tissue and leaf lifespan (Cornelissen et al., 2003; Pérez-Harguindeguy et al., 2016), was calculated as the ratio of fresh leaf area to leaf dry mass (mm^2^ mg^–1^). LDMC was calculated as the ratio of leaf dry mass to leaf fresh mass (mg g^–1^) and is correlated with leaf toughness, resistance to physical hazards, digestibility, and resource-use strategy (Wilson et al., 1999; Cornelissen et al., 2003; Pérez-Harguindeguy et al., 2016). For further statistical analyses, we used the mean values per individual of SLA and LDMC.

Leaf carbon (C) and nitrogen (N) contents were measured on three individuals per species per EcoUnit. Leaf C concentration is related to plant photosynthetic rates and to the amount of structural tissues, while leaf N content is correlated with the N available in the environment and indicates the nutritional quality of the plant (Cornelissen et al., 2003; Pérez-Harguindeguy et al., 2016). Leaves of each individual used for SLA and LDMC measurements were pooled and dried (60°C for at least 72 h). Each pool was then ground individually, and around 1 mg of the ground material per sample was used to measure nutrient concentrations (% dry-leaf mass) and ^15^N signatures (atom ‰) using dry combustion with an elemental analyzer (Euro EA 3000, EuroVector S.p.A; Milano Italy) coupled to a Thermo Delta Vplus isotope ratio mass spectrometer (Thermo Fisher Scientific, Electron, Bremen, Germany).

#### Belowground Trait Measurements at the Species and Community Level

To sample community root traits, two soil cores (10 cm deep, Ø 5 cm) were taken in each half of the EcoUnits (taken at around 35 cm from the edge of the mesocosm), pooled, and sieved (2 mm mesh size). From this homogenized, sieved soil, 45 g were used for nutrient and microbial analyses (see “Abiotic and Biotic Soil Properties”); and the remaining soil was washed over a 63-μm sieve under tap water to collect the roots. Afterward, we discarded the dead roots (i.e., roots without flexibility, hollow, or black) and sorted the rest of them into coarse roots (Ø > 2mm) and fine roots (Ø < 2 mm). The biomass of the different types of roots was measured. Community fine-root trait measurements were performed on a sub-sample of about one eighth [on average 0.42 ± 0.12 g of fresh mass (mean ± sd)] representative of the pool of fine roots collected.

Root traits were measured on two of the five individuals per species sampled for leaf trait measurements. To obtain individual root traits, we dug up a soil cube (L × W × H: 10 × 10 × 10 cm) with the plant basal stem in the center, to excavate the most of the root system of the individual in a standardized way. The soil cubes of two samples from the species *S. laeve* in EcoUnit 1 had a height of around 7 cm, due to the loss of soil at the bottom part. They were included in the analyses as the root system is mainly located in the upper ∼10 cm of the soil, and these samples were not detected as outliers in further analyses. When not processed immediately, soil cubes with roots of plant individuals were stored in the dark in the fridge (4°C) before being processed (maximum 3 days after collection). Soil cubes were soaked in tap water. The soil was then washed over a sieve with a 1 mm mesh, and only roots connected to the base of the plant were collected for further analyses allowing us to confirm the identity of species. The same procedure as the one applied to community roots samples was applied to the roots of the collected individual. Then, from the fine roots sample of each individual, a representative sub-sample of about one eighth [on average 0.43 ± 0.43 g of fresh mass (mean ± sd)] was used to measure fine root traits. When too little root material was collected (which was mainly the case for the species *S. laeve*), we measured fine root traits using the whole pool of fine roots of the individual. Each sub-sample of the fresh fine roots at the community and individual level were scanned with an Epson Perfection 11000XL Scanner (Epson America, Inc., CA, United States) at 600 dpi in grayscale. After scanning, all root samples (coarse, fine, and fine roots scanned) were oven-dried at 60°C for at least 72 h and weighed. Root scans of the community and each individual were analyzed with WinRhizo (Version 2013e Pro, Regent Instruments Inc., Canada), and root traits were calculated for the community and individuals. We measured the root length (cm) at the individual level as well as the specific root length (i.e., the ratio of root length to dry mass of roots (m g^–1^)), root dry matter content (i.e., the ratio of root dry mass to root fresh mass (mg g^–1^)), average root diameter (mm), and root tissue density (i.e., root dry mass divided by fresh root volume; g cm^–3^) at the community level and on two individuals per species per EcoUnit.

The total root length of the individual that was used for further analyses, was calculated as the ratio of the total fresh mass of fine roots multiplied by the root length of the sub-sample and divided by the fresh mass of the sub-sample of fine roots. The shoot:root ratio of two individuals per species per EcoUnit was calculated using the sum of dried aboveground biomass (including leaf mass used for leaf traits measurements) divided by the sum of the dried biomass of all its respective root samples.

### Abiotic and Biotic Soil Properties

From the 45 g of the homogenized and sieved soil used for root community trait measurement, sub-samples were used to measure soil nutrient content, microbial biomass and activity, as well as microbial community structure.

The carbon and nitrogen content (μg) was measured using 10 mg of dried soil (60°C for 72 h), analyzed by dry combustion with an elemental analyzer (Euro EA 3000, EuroVector S.p.A; Milano Italy) coupled to a Thermo Delta Vplus isotope ratio mass spectrometer (Thermo Fisher Scientific, Electron, Bremen, Germany).

We assessed basal respiration (μl O_2_ h^–1^ g^–1^ dry soil), microbial biomass (Cmic; μg C g^–1^ dry soil), and the microbial specific respiratory quotient (qO_2_; μl O_2_ μg^–1^ Cmic h^–1^). These soil microbial properties were measured with 6 g of fresh soil using an O_2_-microcompensation apparatus (Scheu, 1992). The microbial respiratory response was measured every hour during 24 h at 20°C. We measured the respiratory response to the addition of D-Glucose (22 mg per sample added in 1.5 ml deionized water) to calculate substrate-induced respiration. Microbial biomass was calculated from the maximum initial respiratory response, i.e., the lowest average respiratory response of three consecutive hourly measurements within the first 10 h after the first peak caused by the disturbance of the soil during preparation (Beck et al., 1997). The microbial specific respiratory quotient was calculated as the ratio of basal respiration to microbial biomass.

We assessed soil microbial community structure using phospholipid fatty acid composition as described in Frostegård et al. (1991). Briefly, lipids were extracted, fractionated, saponified, and methylated. Fatty acid methyl esters were then transferred into vials, capped, and stored at −20°C until analysis with a gas chromatograph (Clarus 680, Perkin Elmer, Waltham, MA, United States; flame ionization detector; capillary column SP-2560, 100 m × 0.25 mm i.d., 0.2 μm film thickness; carrier gas: helium). We identified fatty acid methyl esters comparing retention times of samples with standards and quantified them calculating phospholipid fatty acid abundances (in nmol g^–1^ dry weight). The entire set of phospholipid fatty acids was used in a multivariate approach to investigate microbial community structure including the biomarkers for Gram-positive bacteria (i15:0; a15:0; i16:0 and i17:0), Gram-negative bacteria (cy17:0), plants (18:1ω9), arbuscular mycorrhizal fungi (16:1ω5; in neutral lipid fraction), and fungi (18:2ω6).

### Statistical Analyses

All statistical analyses and figures were performed and produced with R software version 3.6.3 (R Core Team, 2020), respectively.

Earthworm effects on the total biomass (above- and belowground) and cover of the community, as well as community root traits, were tested using linear models of the package “stats” with Type II F-tests from the package “car” (Fox and Weisber, 2019), with the earthworm treatment as factor and the tree biomass as a covariate. Tree biomass was included as a covariate in models due to its potential effect on plant biomass, cover, and traits *via* the competition for resources (e.g., light and soil nutrients), i.e., this was done to remove the variability in the plant trait values due to the competition with trees. The linear model with a Type II F-test showed that tree biomass was not significantly affected by the earthworm treatment [*F*_(__1_,_10__)_ = 0.06 and *p* = 0.81], and the tree biomass effect on the plant traits was not further discussed. Variables measured at the species or individual level (i.e., species-specific biomass and cover, and functional traits) were tested using linear mixed-effects models with restricted maximum-likelihood (REML) estimates using the “lme4” package (Bates et al., 2014) and Type II Wald Chi-square tests from the package “car” (Fox and Weisber, 2019). The same model structure was used for the different variables: the fixed effects were the tree biomass, the earthworm treatment, the plant species identity, and the interaction of the latest, while the EcoUnit was specified as a random effect. The variance explained by the different linear mixed-effects models (i.e., Conditional *R*^2^, including fixed and random effects) was obtained using the “MuMIn” package (Barton, 2019). Model diagnostics were performed by visual inspection. When necessary, variables were log-transformed (log_10_) to meet the requirements of parametric tests, such as the species biomass, root dry matter content, and root tissue density at the community level, as well as the height, shoot:root ratio, specific leaf area, leaf dry matter content, number of ramets, leaf C:N ratio, root length, root diameter, and specific root length at the individual level. The total cover, species cover, and the proportion of flowering individuals were transformed prior analyses with the logit transformation from the package “car” (Fox and Weisber, 2019). One outlier corresponding to an extreme proportion of flowering individuals of 0.9 for the species *B. ciliatus* in the control treatment (i.e., a proportion around 9 times higher than the one in the other EcoUnits for the same species) was not included in the analysis. Pairwise comparisons with Holm correction were performed by species and by earthworm treatments, when interaction effects of earthworms and species identity were significant, using the package “emmeans” (Lenth et al., 2020).

The effects of earthworms on soil abiotic properties (i.e., carbon and nitrogen content, C:N ratio, and water content) and soil microbial activity (i.e., basal respiration, microbial biomass, microbial specific respiratory quotient) were analyzed using the independent samples *t*-test in case of homoscedasticity or Welch t-statistic in case of heteroscedasticity from the package “stats” (R Core Team, 2020). The effects of earthworms on fatty acid composition of the soil microbial community were analyzed in a multivariate analysis of variance (MANOVA) from the package “stats” (R Core Team, 2020). All figures were made with the package “ggplot2” (Wickham, 2016).

## Results

### Plant Community and Species-Specific Cover and Biomass

**FIGURE 1:**
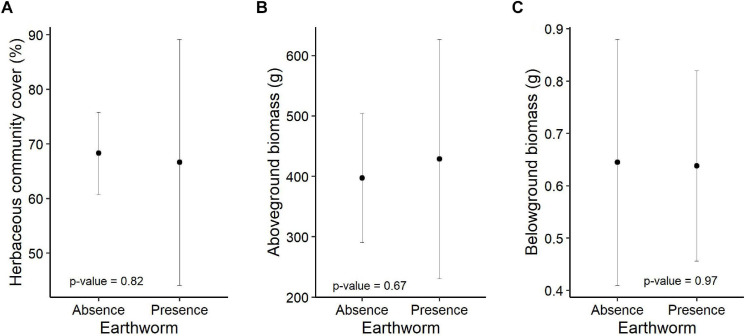
Effect of earthworms on herbaceous community cover **(A)**, aboveground **(B)**, and belowground **(C)** biomass (mean ± sd). The *p*-values of the earthworm treatment are based on linear models. Number of observations per treatment: 6.

The total cover of the herbaceous community, as well as its aboveground and belowground biomass were not significantly affected by earthworm presence [*F*_(__1_,_9__)_ = 0.05, *p* = 0.82, Figure 1A; *F*_(__1_,_9__)_ = 0.20, *p* = 0.67, Figure 1B and *F*_(__1_,_9__)_ = 0.001, *p* = 0.97, Figure 1C, respectively, and Supplementary Material 3] and the biomass of the trees [*F*_(__1_,_9__)_ = 0.44, *p* = 0.52; *F*_(__1_,_9__)_ = 1.51, *p* = 0.25 and *F*_(__1_,_9__)_ = 0.05, *p* = 0.82, respectively].

**FIGURE 2:**
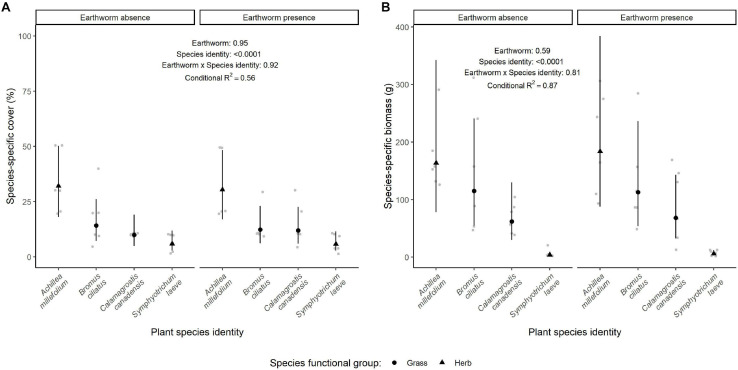
Effect of earthworm presence on species-specific cover **(A)** and biomass **(B)**. Estimated marginal means and confidence intervals are shown in black (after being back-transformed), while data points are included in the background in gray. The conditional *R*^2^ as well as the *p*-values for each factor are based on linear mixed effect models. Number of observations per species and treatment: 6.

The species-specific cover and biomass depended on the species identity (Df = 3, χ^2^ = 53.95, *p* < 0.0001 and Df = 3, χ^2^ = 298.04, *p* < 0.0001, Figures 2A,B, respectively, and Supplementary Material 3), with a higher cover and biomass produced by the species *A. millefolium* and lower values for *S. laeve*. The two grasses species *B. ciliatus* and *C. canadensis* had an intermediate cover and biomass. The species-specific cover and biomass were independent from the presence of earthworms alone (Df = 1, χ^2^ = 0.004, *p* = 0.95 and Df = 1, χ^2^ = 0.29, *p* = 0.59, respectively), and in interaction with the species identity (respectively, Df = 3, χ^2^ = 0.51, *p* = 0.92 and Df = 3, χ^2^ = 0.94, *p* = 0.81), while the tree biomass slightly decreased the species biomass but not the species cover (Df = 1, χ^2^ = 2,88, *p* = 0.09 and Df = 1, χ^2^ = 0.71, *p* = 0.40, respectively).

### Plant Functional Trait Responses at Species and Community Level

**FIGURE 3:**
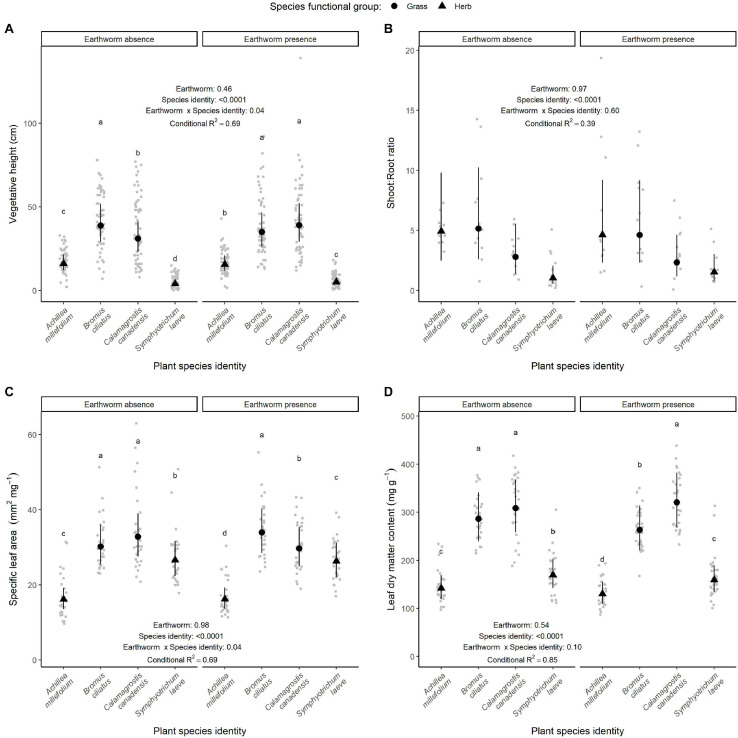
Effect of earthworm presence on plant height **(A)**, shoot:root ratio **(B)**, specific leaf area **(C)**, and leaf dry matter content **(D)**. Estimated marginal means and confidence intervals are shown in black (after being back-transformed), while data points were included in the background in gray. The conditional *R*^2^ as well as the *p*-values for each factor are based on linear mixed effect models. When the interaction between earthworm treatment and species identity were significant, *post hoc* test results are also presented: different letters show significant differences among species within each earthworm treatment. Number of observations per species and treatment: 60 for height; 30 for SLA and LDMC, and 12 for the shoot:root ratio.

**FIGURE 4:**
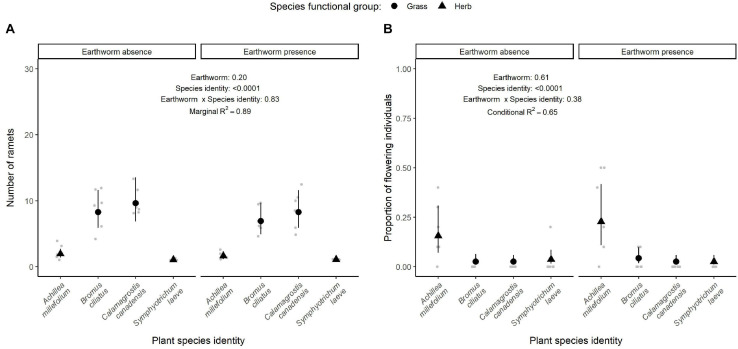
Effect of earthworm presence on reproductive traits: number of ramets **(A)** and proportion of flowering individuals **(B)**. Estimated marginal means and confidence intervals are shown in black (after being back-transformed), while data points were included in the background in gray. The conditional *R*^2^ or marginal *R*^2^ in the case of the number of ramets as well as the *p*-values for each factor are based on linear mixed effect models. Number of observations per species and treatment: 6, except for the proportion of flowering individuals of *B. ciliatus* in the control treatment, where there are five observations.

**FIGURE 5:**
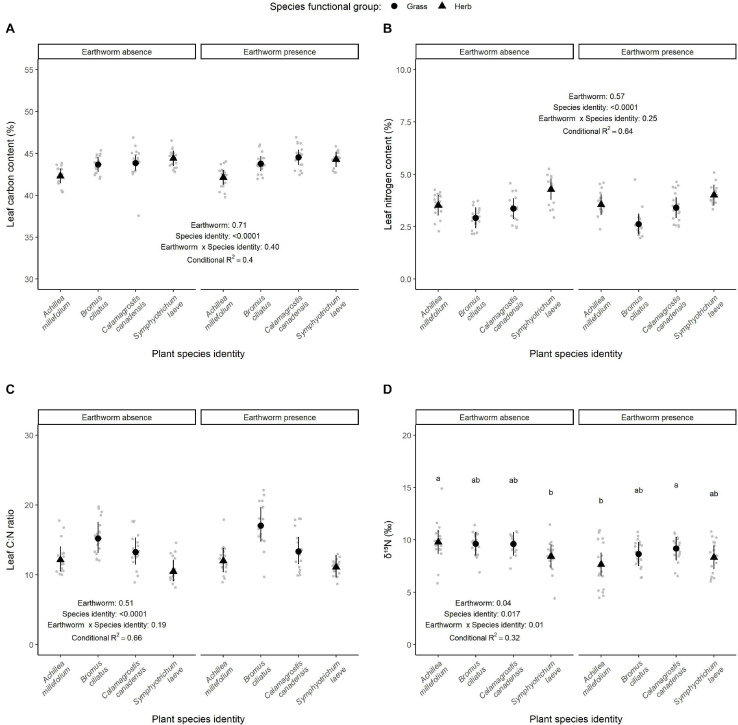
Effect of earthworm presence on leaf carbon **(A)** and nitrogen **(B)** content as well as their C/N ratio **(C)** and δ^15^N signature **(D)**. Estimated marginal means and confidence intervals are shown in black (after being back-transformed), while data points were included in the background in gray. The conditional *R*^2^ as well as the *p*-values for each factor are based on linear mixed effect models. When the interaction between earthworm treatment and species identity were significant, *post hoc* test results are also presented: different letters show significant differences among species within each earthworm treatment. Number of observations per species and treatment: 18, except for *S. leave* in the control treatment, where there are 17 observations.

**FIGURE 6:**
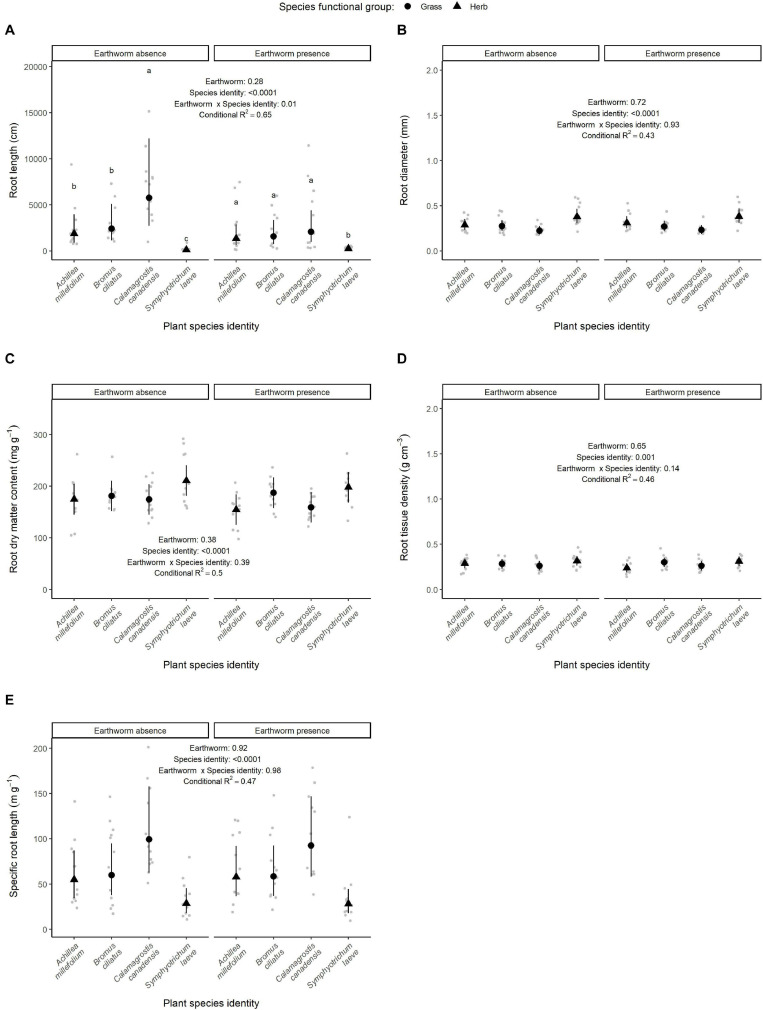
Effect of earthworm presence on root length **(A)**, root diameter **(B)**, root dry matter content **(C)**, root tissue density **(D)**, and specific root length **(E)**. Estimated marginal means and confidence intervals are shown in black (after being back-transformed), while data points were included in the background in gray. The conditional *R*^2^ as well as the *p*-values for each factor are based on linear mixed effect models. When the interaction between earthworm treatment and species identity were significant, *post hoc* test results are also presented: different letters show significant differences among species within each earthworm treatment. Number of observations per species and treatment: 12.

Among the aboveground traits studied, all of them depended on species identity and were negligibly affected by the tree biomass (Table 1 and Supplementary Material 3). Four out of the 15 above- and belowground traits measured were significantly affected by earthworm presence. Although earthworm presence did not affect the shoot:root ratio (Figure 3), nor the proportion of flowering individuals and number of ramets (Figure 4) and the nutrient content of the plants (Figure 5), it induced changes in the plant height, leaf (Figure 3) and roots traits (Figure 6).

**TABLE 1:** Summary of the models on the effects of earthworms on the different plant functional traits (χ^2^, *p*-value, and conditional or marginal *R*^2^ in the case of the number of ramets).

	Tree biomass		Species identity (S)		Earthworm (E)		Interaction S × E		
	χ^2^	*p*-Value	χ^2^	*p*-Value	χ^2^	*p*-Value	χ^2^	*p*-Value	*R*^2^
**(1) Aboveground traits**									
Height	**3.97**	**0.05**	**992.46**	**<0.0001**	0.54	0.46	**8.30**	**0.04**	0.69
Shoot:root ratio	0.07	0.79	**46.15**	**<0.0001**	0.001	0.97	1.86	0.60	0.39
Specific leaf area	2.44	0.12	**419.69**	**<0.0001**	0.00	0.98	**8.29**	**0.04**	0.69
Leaf dry matter content	2.31	0.13	**1181.19**	**<0.0001**	0.37	0.54	6.25	0.10	0.85
Number of ramets	0.03	0.86	**393.90**	**<0.0001**	1.62	0.20	0.90	0.83	0.89
Proportion of flowering individuals	0.04	0.84	**69.37**	**<0.0001**	0.25	0.61	3.11	0.38	0.65
**(2) Nutrient traits**									
Leaf carbon content	0.16	0.69	**70.01**	**<0.0001**	0.14	0.71	2.96	0.40	0.40
Leaf nitrogen content	3.51	0.06	**150.03**	**<0.0001**	0.33	0.57	4.07	0.25	0.64
Leaf C:N ratio	3.12	0.08	**165.51**	**<0.0001**	0.44	0.51	4.74	0.19	0.66
Leaf δ^15^N signature	0.83	0.36	**10.17**	**0.017**	**4.31**	**0.04**	**10.56**	**0.01**	0.32
**(3) Belowground traits**									
Root length	1.36	0.24	**153.13**	**<0.0001**	1.19	0.28	**10.75**	**0.01**	0.65
Root diameter	0.47	0.49	**55.57**	**<0.0001**	0.12	0.72	0.44	0.93	0.43
Root dry matter content	**4.50**	**0.03**	**31.06**	**<0.0001**	0.77	0.38	2.99	0.39	0.50
Root tissue density	**6.98**	**0.01**	**16.09**	**0.001**	0.21	0.65	5.40	0.14	0.46
Specific root length	3.34	0.07	**62.27**	**<0.0001**	0.01	0.92	0.16	0.98	0.47

*Statistical differences are based on linear mixed effect models with earthworm treatment and species identity as fixed effects alone and in interaction, the tree biomass as a covariate, and the EcoUnits specified as a random effect. Significant effects are highlighted in bold.*

Earthworm effects on plant height depended on the species identity as shown by the significant interaction between plant species identity and earthworm treatment (*p* = 0.04, Table 1 and Figure 3). Plant height was also negatively affected by tree biomass, especially for the species *S. laeve* and *C. canadensis* (Supplementary Material 4). Overall, the height of *B. ciliatus* (mean ± sd: 40.78 ± 17.58 cm) and *C. canadensis* (mean ± sd: 39.92 ± 20.53 cm) were more than twice higher than the one of *A. millefolium* (mean ± sd: 17.64 ± 6.95 cm) and *S. laeve* (mean ± sd: 5.97 ± 3.93 cm). *Bromus ciliatus* and *C. canadensis* showed different heights in the control treatment, but this difference disappeared in the presence of earthworms (Figure 3 and Supplementary Material 5), where we observed on average an increase in *C. canadensis*’ height of around 7 cm and a decrease in *B. ciliatus*’ height of around 5 cm.

Leaf trait values (i.e., specific leaf area and leaf dry matter content) depended also on the interaction between species identity and the presence of earthworms, although this interaction was only marginally significant for leaf dry matter content (*p* = 0.04 and *p* = 0.10, respectively; Figure 3). Although the two grass species showed similar SLA and LDMC in the control treatment, they differed in the presence of earthworms (Supplementary Material 5): *B. ciliatus* showed a higher specific leaf area (+ 13.3%) and a lower leaf dry matter content (−8.8%) in the presence of earthworms than in the control treatment, while these traits responded contrarily in *C. canadensis* (−10.2% and +2.1%, respectively). The two herbs species *S. laeve* and *A. millefolium* differed in their specific leaf area and leaf dry matter content, independent of the earthworm treatment.

Leaf carbon and nitrogen content and the leaf C:N ratio did not change in response to the earthworm treatments and the interaction between species identity and earthworm treatments, but varied across species (Table 1 and Figure 5). The lowest carbon and nitrogen content were measured in *A. millefolium* and *B. ciliatus*, respectively. *Bromus ciliatus* showed the highest C:N ratio, while *S. laeve* had the lowest. Tree biomass tended to increase leaf nitrogen content (*p* = 0.06), but tended to decrease the C:N ratio (*p* = 0.08), while it did not affect the carbon content (*p* = 0.69, Table 1). However, the δ^15^N signature in plants depended on the interaction between earthworm presence and species identity (*p* = 0.014, Figure 5 and Table 1). Overall, we observed a decrease of the δ^15^N signature in plant tissues when earthworms were present (−9.6%, *p* = 0.04, Table 1). However, this effect was mainly observed for the species *A. millefolium* (−21.5%, *p* = 0.001, Supplementary Material 5).

Regarding the root traits, the root average diameter, root dry matter content, root tissue density, and specific root length at the community level did not vary significantly according to the earthworm treatment [*F*_(__1_,_9__)_ = 0.49, *p* = 0.50; *F*_(__1_,_9__)_ = 0.46, *p* = 0.51; *F*_(__1_,_9__)_ = 0.0002, *p* = 0.98 and *F*_(__1_,_9__)_ = 0.65, *p* = 0.44, respectively]. However, the root tissue density of the community decreased with an increase of the tree biomass [*F*_(__1_,_9__)_ = 5.50, *p* = 0.04], while the specific root length was marginally positively affected by tree biomass [*F*_(__1_,_9__)_ = 3.52, *p* = 0.09].

At the species level, root traits varied across species and changed with tree biomass. On average, the species *S. laeve* showed a larger diameter and a higher root dry matter content than *A. millefolium*, *B. ciliatus*, and *C. canadensis*. The lowest values of these three traits were measured on the species *C. canadensis*, as well as *A. millefolium* regarding the dry matter content. *Achillea millefolium*, *B. ciliatus*, and *C. canadensis* showed similar root tissue density, but this trait was higher in *S. laeve*, although it did not significantly differ from the one of *B. ciliatus*. *Calamagrostis canadensis* showed the highest values of specific root length, while the lowest were measured on *S. laeve*. *Achillea millefolium* and *B. ciliatus* showed similar intermediate values of specific root length. Higher tree biomass also decreased the root tissue density (*p* = 0.008) at the species level except for the species *B. ciliatus*, and overall the root dry matter content (*p* = 0.03) of the different species (Supplementary Material 4).

The total root length differed between species (*p* < 0.0001, Table 1 and Figure 6) with *S. laeve* showing on average the lowest and *C. canadensis* the highest. Although we found little evidence of earthworm effects on the root traits, the total root length was affected by the interaction between earthworm and species identity (*p* = 0.01, Figure 6). The total length of the root produced by *C. canadensis* was significantly lower in the presence of earthworms than in their absence (Figure 6 and Supplementary Material 4). Furthermore, without earthworms, the total length of the roots produced by *C. canadensis* was significantly higher than the one from *A. millefolium* and *B. ciliatus*, while the species *S. laeve* showed a total length lower than the three other species. However, the difference between the grass species was weakest in the presence of earthworms (Figure 6 and Supplementary Material 5): the root length of *C. canadensis* decreased (−44.3%) until reaching a similar root length to the one of *B. ciliatus* and *A. millefolium*.

### Earthworm Effects on Soil Abiotic Parameters and Microbes

As earthworm effects on plants could be mediated via changes in soil abiotic and biotic conditions, we explored soil nutrient concentrations and microbial properties. Overall, *t*-test results showed that the presence of earthworms did not affect soil basal respiration (Df = 10; *t* = 0; *p* = 1), microbial biomass (Df = 10; *t* = −0.34; *p* = 0.74), microbial specific respiratory quotient (Df = 10; *t* = 1.17; *p* = 0.27), water content (Df = 10; *t* = 0.59; *p* = 0.57), and nutrient content (Carbon: Df = 10; *t* = −0.11; *p* = 0.92; Nitrogen: Df = 10; *t* = −0.54; *p* = 0.60 and C/N ratio: Df = 10; *t* = 0.88; *p* = 0.40). The microbial community structure was also not significantly affected by the presence of earthworms (Df = 1; *F* = 1.48; *p* = 0.41).

## Discussion

### Plant Community and Trait Responses to Earthworm Presence

Although earthworms did not induce significant changes in plant cover and biomass, both at the community- and species-level (hypothesis i), they influenced some of the assessed plant functional traits. More specifically, four out of the fifteen above- and belowground traits were significantly affected by the interaction between earthworm presence and plant species identity. These plant species- and trait-specific responses to earthworm presence are subtle but may alter competitive interactions between plant species and should thus be further explored as potential mechanism underlying widely observed plant community changes in response to earthworm invasion (e.g., Hale et al., 2008; Craven et al., 2017).

Several studies have shown that exotic and native earthworms stimulate the productivity of different plant species and communities; but also neutral effects were observed (Scheu and Parkinson, 1994; Scheu, 2003; Dávalos et al., 2013; van Groenigen et al., 2014). Mechanisms/pathways involving plant functional traits behind these inconsistent results are not well known, but a meta-analysis showed that native earthworm effects become weak to non-significant the more fertile the soil is and with a high proportion of sand in the soil (van Groenigen et al., 2014). However, so far, few studies showed a modification of plant functional traits other than biomass-related traits with native or exotic earthworm presence (e.g., Wurst et al., 2003; Hale et al., 2008; Eisenhauer et al., 2009a; Laossi et al., 2009; Dávalos et al., 2013, 2015; Cameron et al., 2014; Dobson et al., 2017; Agapit et al., 2018; Blume-Werry et al., 2020; Liu et al., 2020).

With four of the studied traits that responded significantly to earthworms presence, our study partly confirmed our hypothesis that earthworms affect the traits related to plant resource uptake and development (hypothesis ii). Aboveground traits, such as the vegetative height, specific leaf area, and leaf dry matter content responded to earthworm presence, although the responses were plant species-specific (hypothesis iii). This study thus confirms results from previous studies that earthworms can impact plant height (Haimi and Einbork, 1992; Eisenhauer et al., 2009b; Dávalos et al., 2013, 2015; Agapit et al., 2018) and demonstrates that earthworms could affect plant resource acquisition and competitive abilities by inducing changes in leaf traits, which have not been previously described. However, we did not observe any significant effect of earthworm presence on the reproductive plant traits (i.e., the proportion of flowering individuals and number of ramets). Diverse results were observed in previous studies. For example, Dávalos et al. (2013) also showed that the probability of flowering was not affected by exotic earthworms, while the number of culms produced by sedges decreased. By contrast, Blume-Werry et al. (2020) showed positive and neutral effects of exotic earthworm presence on the production of floral shoots by native plants, while Zaller and Arnone (1999) measured a higher number of ramets produced by native plant species close to native earthworm casts. Similar to plant biomass responses, earthworm effects on reproductive plant traits may vary with the abiotic and biotic context though. For instance, Eisenhauer and Scheu (2008) observed neutral effects of native earthworms on the number of legume flower heads in the absence of grasses, while earthworms reduced their number in the presence of grasses by fostering the competitive strength of grasses against legumes. Other biotic factors such as the composition and diversity of the earthworm community (e.g., Hale, 2004; Craven et al., 2017) could also have differently affected the trait responses of the plant species studied.

In the present study, earthworms did not enhance the N content of the plant species, which is in line with some previous studies with native earthworms (Wurst et al., 2005) and a meta-analysis (van Groenigen et al., 2014), but is in contrast to some others showing that earthworms increase nitrogen concentrations in grass shoots (Wurst et al., 2005; Eisenhauer and Scheu, 2008). An increase in leaf tissue nitrogen concentrations could also have been expected, as earthworm activity (e.g., enhanced nitrogen mineralization) and also dead/decomposing earthworms would increase plant-available nitrogen (Barois et al., 1999; Kim et al., 2017; Kos et al., 2017). The lack of change in leaf nutrient content may be linked to the few changes in root traits between earthworm treatments, as roots have an important role in nutrient uptake and transfer to aboveground organs (Oaks and Hirel, 1985; Eissenstat, 1992; Bloom et al., 2003; Brown et al., 2004; Peel, 2013). Surprisingly, although earthworms are known to decrease the spatial homogeneity of soil resource distribution (Shuster et al., 2001), we did not observe a strong modification of the plant belowground traits at the species and community level, which differed from what was shown in previous studies. Blume-Werry et al. (2020) showed that fine root growth of the plant community was higher in the presence of exotic earthworms in meadows after 55 days and after 103 days of experiment independent of the vegetation type (meadows vs. heath, with plant communities mainly composed of forbs or shrubs, respectively, and grasses) than in the absence of earthworms. Cameron et al. (2014) showed that the proportion of *A. millefolium* roots present in exotic earthworms burrows was important during the first month of growth, but root growth in soil cracks became similarly important after 2 and 3 months. These authors also observed an opposite pattern for the root distribution of *Campanula rotundifolia*: although its root occurrence was similar in cracks and burrows after 1 month of experiment, it decreased in exotic earthworm burrows after 2 and 3 months (Cameron et al., 2014). Thus, the minor responses of root traits to earthworms observed in the present study could be explained by potential transient effects, which may also be plant species-specific due to differences in the growth rate and growth strategy. Thus, root trait changes induced by earthworms could have taken place at earlier stages of plant growth, with a difference between treatments being less pronounced after 3 months of experiment. Future studies may thus explore potential temporal dynamics of earthworm effects on plant traits. Another explanation for minor root trait responses could be that soil nutrients (e.g., nitrogen) were not limiting enough to induce different responses of the root traits between the two treatments. Thus, plants which could preferentially forage in the burrows of earthworms would not take any advantage of doing this against plants that would not express this behavior, and thus that earthworm effects on root foraging in burrows would be more visible under limiting soil nutrient levels (van Groenigen et al., 2014). This could also explain why we did not observe any significant changes in the nutrient content and microbial community properties in the upper soil layer in response to earthworms, which is in contrast to previous meta-analyses (Ferlian et al., 2018, 2020). Invasive earthworms were shown to have opposing effects on organic and mineral soil layers for carbon and nitrogen concentrations (Ferlian et al., 2020), and their effects on soil microorganisms were neutral when the different soil layers were pooled (Ferlian et al., 2018). Although we cannot exclude that the duration of the experiment may have been too short to observe the effect of earthworms in the large volume of soil (∼1 m^3^), the analysis of soil properties across all soil layers could also have masked the effect of earthworms on the nutrient content and microbial community properties.

Other root traits could also be investigated in further studies. Mycorrhizal associations deserve some attention, as they play a key role in plant development and nutrient acquisition and were shown to be significantly affected by invasive earthworms (Lawrence et al., 2003; Paudel et al., 2016). The abilities of plants to spread their root system laterally or their rooting depth may also have some importance in the belowground plant response to earthworm presence (Eisenhauer et al., 2012b). Species with a higher density of roots in the upper soil layer or a strong lateral spread of their roots might take advantage of being closer to earthworm casts and middens at the soil surface that are known to affect plant growth. For example, studies showed that the proximity of the plant to native earthworm casts enhanced plant growth and ramet production (Zaller and Arnone, 1999), which could be explained by the presence of hormone-like molecules regulating plant growth in earthworm casts (Brown et al., 2004; Puga-Freitas et al., 2012).

### More Pronounced Responses of Grasses Than of Herbs to Earthworm Presence

Our study demonstrated that earthworm effects on plant functional traits were plant species-specific. Although we only tested two grass and two herb species in the present study, earthworm effects tended to be stronger in grasses than in herbs (hypothesis iii). The more pronounced response of grasses to earthworm presence can be explained by their rapid growth and their high resource competition abilities under high resource conditions (Linder et al., 2018), such as those observed under earthworms presence (Eisenhauer and Scheu, 2008).

Interestingly, however, the two grass species studied showed opposite responses to earthworms. Earthworms induced an increase of *C. canadensis* height and leaf dry matter content, while it decreased its specific leaf area and total root length. A possible explanation for this result is a change in *C. canadensis*’ growth strategy due to the potential changes in the spatial distribution of soil nutrients resulting from earthworm activity. More precisely, *C. canadensis* could have shifted its biomass allocation from root production toward apical growth and more structural/less flimsy tissues, rather than into the lateral spread (i.e., ramet production) or reproduction (i.e., flower production) that did not change with earthworm presence. However, although this species showed a high leaf ^15^N signature that was not affected by earthworm presence, its leaf ^15^N signature was slightly positively correlated to the height in earthworm presence only (Supplementary Material 6), which suggests that *C. canadensis* could take advantage of the nutrients coming from the litter decomposed by earthworms or found in their casts. This result is also corroborated by the leaf ^15^N signature of this species, which was negatively correlated to its specific root length. The increase of the leaf ^15^N signature when the cost of constructing roots decreased and root systems were less developed (i.e., low SRL) suggests that *C. canadensis* could preferentially forage in the nutrient-rich burrows or casts of earthworms. Notably, although we did not observe any significant earthworm effects on soil N content at the end of the study, this gross measurement would not be able to reflect the concentrations of plant-available ammonium and nitrate as well as potential temporal or spatial differences in nutrient availability in response to earthworm activity.

In contrast to *C. canadensis*, *B. ciliatus* showed a higher specific leaf area and a lower leaf dry matter content in the presence of earthworms, while its height, ramet, and flower production, and root traits were unaffected by earthworms. This species probably directed its resources toward the production of a more costly and dense root system (i.e., with a higher root diameter, dry matter content, and tissue density) instead of favoring its aboveground growth or investing into reproduction. This shift of resource allocation to the root system was emphasized in presence of earthworms where plant height and shoot:root ratio were significantly correlated with root dry matter content and root tissue density (Supplementary Material 6). Furthermore, we did not observe any significant correlation between the δ^15^N signature of *B. ciliatus* and its height or its shoot:root ratio nor any other traits in the presence of earthworms (Supplementary Material 6). To sum up, our results indicate that *B. ciliatus* did not preferentially forage in earthworm burrows or casts, and that its development and thus changes of leaf traits were rather independent of nutrients coming from the litter decomposed by earthworms or their casts. This result is in line with the observation made by Zaller and Arnone (1999), who did not observe any significant relationship between native earthworm activity (i.e., surface cast production) and the growth (i.e., production of ramets) of the native congeneric species *B. erectus*. Thus, grasses were shown to respond differently to the presence of earthworms, even if both showed stronger responses than herbs in the present study.

Indeed, among the measured traits, only the δ^15^N signature in *A. millefolium* was significantly affected by earthworm presence. However, the two herbs species studied showed opposite responses to earthworm presence. While we observed a strong decrease of the δ^15^N signature in leaves of *A. millefolium* when earthworms were present, this value was not correlated to any of the other traits studied (Supplementary Material 6). As *A. millefolium* has been reported to preferentially forage in exotic earthworms burrows (Cameron et al., 2014), we would have expected to observe an effect of earthworms on various root traits as well as significant correlations between these root traits (i.e., SRL) and the δ^15^N signature in leaves when earthworms were present. Our results were thus unexpected and could potentially be related to the fact that exotic earthworms can also increase the leaching of soil nutrients through their burrows that function as preferential flow pathways of soil surface water (Ferlian et al., 2020). This phenomenon is particularly pronounced for the vertical burrows of anecic earthworms like *L. terrestris* that dominated the earthworm community in the present study.

In contrast to *A. millefolium*, the development of *S. laeve* was correlated to its root traits. This species overall invested more of its resources into the production of belowground costly tissue, such as roots with large root diameter and high dry matter content, than in its aboveground development. This response was found to be most accentuated in the presence of earthworms as indicated by the positive correlation between the δ^15^N signature with the root diameter of *S. laeve* in the presence of earthworms, as well as by the negative correlation of the δ^15^N signature with its ramet production, and the non-significant although marginally positive correlation with its height (Supplementary Material 6). Furthermore, the negative correlation between δ^15^N signature in *S. laeve* in the presence of earthworms and its specific root length also suggests that this species could preferentially forage in the nutrient-rich burrows or casts of earthworms. However, the consequences of this strategy in response to earthworms remain to be investigated in long-term studies and under natural field conditions.

## Conclusion

Our study provides novel insights into the trait responses of different plant species under earthworm invasion and indicates that these changes of plant strategies are not necessarily mediated by the effects of earthworms on gross soil abiotic properties and on the soil microbial activity or community structure. These results advance our understanding of the mechanisms behind plant community changes in response to earthworm invasion. Plant responses to earthworms were species-specific, and the development strategies of grass species were observed to be more likely affected by earthworm presence than that of herbs. However, even if the grass species responded faster to earthworm presence, herb responses to invasive earthworms may only be pronounced with time, calling for long-term studies. Indeed, the slow development due to the high costs of tissue production in slower-growing species may have masked potential beneficial effects of earthworms on their development in our experiment, which may have been too short to reflect potential beneficial effects.

Nevertheless, our results suggest that by modifying some plant strategies, earthworms could potentially change the outcome of interspecific competition between plants in the longer term and may lead to a shift in plant species and functional group dominance in the plant community, as commonly observed in field studies on plant community responses to earthworm invasion (e.g., Craven et al., 2017). Thus, the strong adaptability and trait responses of the grass species to earthworm presence could explain why grasses commonly benefit in the early stages of earthworm invasion and the associated plant community changes (Craven et al., 2017). However, the potential long-term effects of earthworms on plant traits and strategies remain to be investigated, as we showed that earthworms may also have beneficial effects on the resource uptake strategies of some herb species. Furthermore, the observed opposite patterns of response within each of the two plant functional groups in the presence of earthworms, suggest that the use of plant functional groups to characterize the consequences and mechanisms of earthworm invasion for plant community composition may mask some important differences and that approaches at the species level should be preferred in future long-term studies. Although with four plant species, we were unable to rigorously test this concept in the present work, the plant economics spectrum (Reich, 2014) may provide a powerful conceptual backbone to guide hypotheses of future research on invasive earthworm effects on native plant species’ strategies and communities.

## Data Availability Statement

The datasets presented in this study can be found in online repositories. The names of the repository/repositories and accession number(s) can be found below: iDiv Data Repository and https://doi.org/10.25829/idiv.1918-3-3293.

## Author Contributions

LT, MT, MPT, and NE conceived the study. LT, MT, TK, OF, and MPT set-up the study. LT, TK, MT, OF, and AL contributed to the data collection and processing. LT and OF analyzed the data with substantial inputs from RB. LT wrote the manuscript with substantial inputs from NE and OF. All authors contributed to the article and approved the submitted version.

## Conflict of Interest

The authors declare that the research was conducted in the absence of any commercial or financial relationships that could be construed as a potential conflict of interest. The handling editor declared a past co-authorship with the authors, OF, MPT, and NE.
