# Supplementary material for: Do Invasive Earthworms Affect the Functional Traits of Native Plants?
Source: Front Plant Sci. 2021 Mar 16;12:627573. doi: 10.3389/fpls.2021.627573 (PMC8007962; doi:10.3389/fpls.2021.627573)
Supplement: Supplementary file 1 [file Data_Sheet_1.docx]

**Supplementary material 1**

**Do invasive earthworms affect the functional traits of native plants?**

**Lise Thouvenot^1,2*^, Olga Ferlian^1,2^, Remy Beugnon^1,2^, Tom Künne^1,2^, Alfred Lochner^1,2^, Madhav P. Thakur^1,2,3^, Manfred Türke^1,2^, and Nico Eisenhauer^1,2^**

^1^German Centre for Integrative Biodiversity Research (iDiv) Halle-Jena-Leipzig, Leipzig, Germany

^2^Institute of Biology, Leipzig University, Leipzig, Germany

^3^Terrestrial Ecology Group, University of Bern, Bern, Switzerland.

* **Correspondence:**

Lise Thouvenot

[lise.thouvenot@idiv.de](mailto:lise.thouvenot@idiv.de)


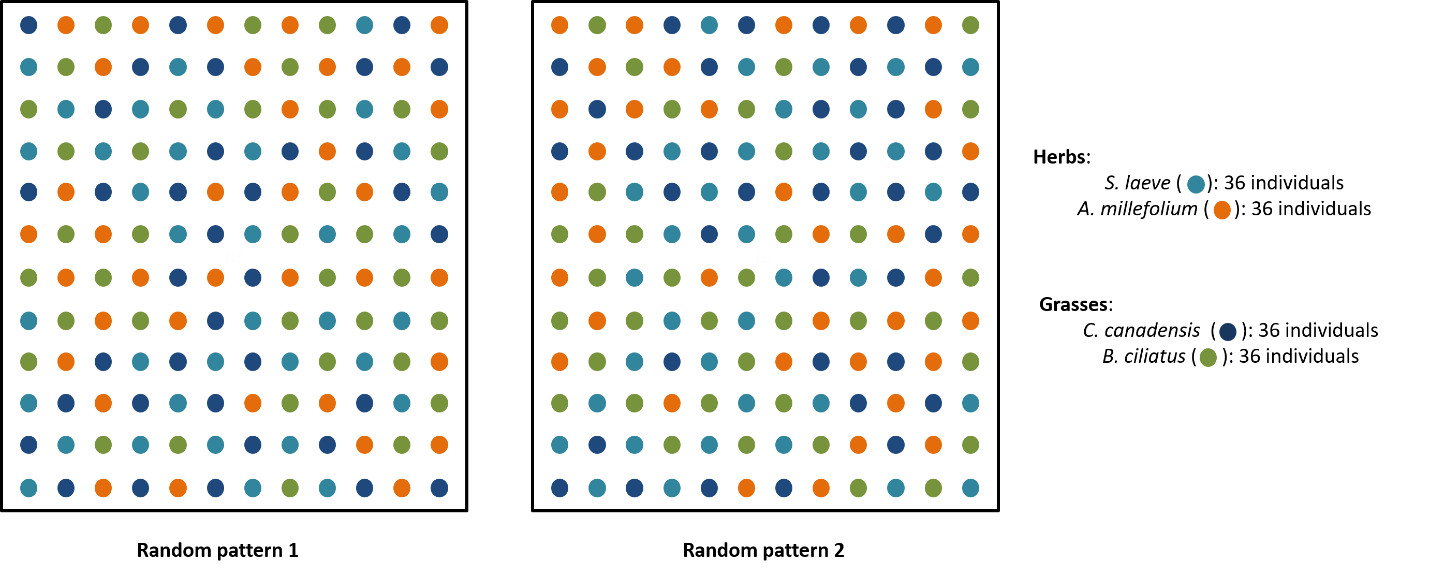


**Figure 1:** Schema of the two plant community patterns. These patterns were set up to introduce heterogeneity in the location of the different plant species of the plant understory community, keeping the number of individuals per species and per EcoUnit constant (grid of 12 by 12 individuals, with 36 individuals per species, thus 144 individuals per community). In both patterns, the plant community consisted of 50% grasses and 50% herbs, with herbs and grasses planted in an alternate order. Species within functional groups were randomly distributed to functional group locations in each pattern.
