# Supplementary material for: Do Invasive Earthworms Affect the Functional Traits of Native Plants?
Source: Front Plant Sci. 2021 Mar 16;12:627573. doi: 10.3389/fpls.2021.627573 (PMC8007962; doi:10.3389/fpls.2021.627573)
Supplement: Supplementary file 2 [file Data_Sheet_2.docx]

**Supplementary material 2**

**Do invasive earthworms affect the functional traits of native plants?**

**Lise Thouvenot^1,2*^, Olga Ferlian^1,2^, Remy Beugnon^1,2^, Tom Künne^1,2^, Alfred Lochner^1,2^, Madhav P. Thakur^1,2,3^, Manfred Türke^1,2^, and Nico Eisenhauer^1,2^**

^1^German Centre for Integrative Biodiversity Research (iDiv) Halle-Jena-Leipzig, Leipzig, Germany

^2^Institute of Biology, Leipzig University, Leipzig, Germany

^3^Terrestrial Ecology Group, University of Bern, Bern, Switzerland.

* **Correspondence:**

Lise Thouvenot

[lise.thouvenot@idiv.de](mailto:lise.thouvenot@idiv.de)

**Table 1:** Summary of the statistical models on the effects of the different experimental factors on the number of plant individuals (total number and species-specific). Statistical differences of the total abundance are based on linear model with earthworm treatment and tree productivity as explanatory variables. Statistical differences of the species-specific abundance are based on linear mixed effect model with tree biomass, earthworm treatment and species identity as well as the interaction between earthworm treatment and species identity as fixed effects, and EcoUnit specified as a random effect.

| Total abundance | | | | Species-specific abundance | | | |
| --- | --- | --- | --- | --- | --- | --- | --- |
|  | Df | F | p-value |  | Df | χ^2^ | p-value |
| Tree biomass | 1 | 1.29 | 0.29 | Tree biomass | 1 | 1.28 | 0.26 |
| Earthworm (E) | 1 | 0.008 | 0.93 | Earthworm (E) | 1 | 0.008 | 0.93 |
| Species identity (S) | / | | | Species identity (S) | 3 | 0.54 | 0.91 |
| Interaction S x E | / | | | Interaction S x E | 3 | 0.55 | 0.91 |
