# Supplementary material for: Do Invasive Earthworms Affect the Functional Traits of Native Plants?
Source: Front Plant Sci. 2021 Mar 16;12:627573. doi: 10.3389/fpls.2021.627573 (PMC8007962; doi:10.3389/fpls.2021.627573)
Supplement: Supplementary file 3 [file Data_Sheet_3.docx]

**Supplementary material 3**

**Do invasive earthworms affect the functional traits of native plants?**

**Lise Thouvenot^1,2*^, Olga Ferlian^1,2^, Remy Beugnon^1,2^, Tom Künne^1,2^, Alfred Lochner^1,2^, Madhav P. Thakur^1,2,3^, Manfred Türke^1,2^, and Nico Eisenhauer^1,2^**

^1^German Centre for Integrative Biodiversity Research (iDiv) Halle-Jena-Leipzig, Leipzig, Germany

^2^Institute of Biology, Leipzig University, Leipzig, Germany

^3^Terrestrial Ecology Group, University of Bern, Bern, Switzerland.

* **Correspondence:**

Lise Thouvenot

[lise.thouvenot@idiv.de](mailto:lise.thouvenot@idiv.de)

**Table 1:** Model results for the effects of tree biomass and earthworms (*i.e.* “ew_presenceNoEW”) on the total cover of the plant community, plant above- and belowground biomass, as well as plant community root traits (*i.e.* specific root length, root diameter, root tissues density, and root dry matter content). Data were analyzed using linear models with earthworm treatment and tree productivity as explanatory variables. The transformation applied on the data as well as the sample size are also presented.

| **Response** | **n** | **Transformation** | **Explanatory** | **Estimate** | **SE** | **t-value** |
| --- | --- | --- | --- | --- | --- | --- |
| Total cover | 12 | logit | (Intercept) | 1.69 | 1.26 | 1 |
|  |  |  | tree_biomass | 0.00 | 0.00 | -1 |
|  |  |  | ew_presenceNoEW | -0.12 | 0.51 | 0 |
| Aboveground biomass | 12 | none | (Intercept) | 688.66 | 221.10 | 3 |
|  |  |  | tree_biomass | -0.39 | 0.31 | -1 |
|  |  |  | ew_presenceNoEW | -40.16 | 89.97 | 0 |
| Belowground biomass | 12 | none | (Intercept) | 0.71 | 0.31 | 2 |
|  |  |  | tree_biomass | 0.00 | 0.00 | 0 |
|  |  |  | ew_presenceNoEW | 0.00 | 0.13 | 0 |
| Community specific root length | 12 | none | (Intercept) | 44.63 | 33.66 | 1 |
|  |  |  | tree_biomass | 0.09 | 0.05 | 2 |
|  |  |  | ew_presenceNoEW | -11.08 | 13.70 | -1 |
| Community root diameter | 12 | none | (Intercept) | 0.25 | 0.03 | 8 |
|  |  |  | tree_biomass | 0.00 | 0.00 | -1 |
|  |  |  | ew_presenceNoEW | 0.01 | 0.01 | 1 |
| Community root dry matter content | 12 | log10 | (Intercept) | 2.31 | 0.09 | 25 |
|  |  |  | tree_biomass | 0.00 | 0.00 | -2 |
|  |  |  | ew_presenceNoEW | 0.03 | 0.04 | 1 |
| Community root tissue density | 12 | log10 | (Intercept) | -0.44 | 0.07 | -6 |
|  |  |  | tree_biomass | 0.00 | 0.00 | -2 |
|  |  |  | ew_presenceNoEW | 0.00 | 0.03 | 0 |

**Table 2:** Model results for the effects of earthworms, species identity, and tree biomass on plant species-specific cover and biomass. Data were analyzed using linear mixed effect models with tree biomass, earthworm treatment and species identity as well as the interaction between earthworm treatment and species identity as fixed effects, and EcoUnit specified as a random effect. The transformation applied on the data as well as the sample size are also presented.

| **Response** | **n** | **Transformation** | **Explanatory** | **Estimate** | **SE** | **t-value** |
| --- | --- | --- | --- | --- | --- | --- |
| Species-specific cover | 48 | logit | (Intercept) | -2.35 | 0.62 | -4 |
|  |  |  | tree_biomass | 0.00 | 0.00 | -1 |
|  |  |  | ew_presenceNoEW | 0.03 | 0.41 | 0 |
|  |  |  | species_idAM | 1.97 | 0.39 | 5 |
|  |  |  | species_idBC | 0.83 | 0.39 | 2 |
|  |  |  | species_idCC | 0.81 | 0.39 | 2 |
|  |  |  | ew_presenceNoEW:species_idAM | 0.05 | 0.55 | 0 |
|  |  |  | ew_presenceNoEW:species_idBC | 0.14 | 0.55 | 0 |
|  |  |  | ew_presenceNoEW:species_idCC | -0.24 | 0.55 | 0 |
| Species-specific biomass | 48 | log10 | (Intercept) | 1.25 | 0.31 | 4 |
|  |  |  | tree_biomass | 0.00 | 0.00 | -2 |
|  |  |  | ew_presenceNoEW | -0.18 | 0.17 | -1 |
|  |  |  | species_idAM | 1.49 | 0.14 | 11 |
|  |  |  | species_idBC | 1.28 | 0.14 | 9 |
|  |  |  | species_idCC | 1.06 | 0.14 | 8 |
|  |  |  | ew_presenceNoEW:species_idAM | 0.13 | 0.20 | 1 |
|  |  |  | ew_presenceNoEW:species_idBC | 0.18 | 0.20 | 1 |
|  |  |  | ew_presenceNoEW:species_idCC | 0.13 | 0.20 | 1 |

**Table 3:** Model results for the effects of earthworms, species identity, and tree biomass on aboveground plant traits (*i.e.* height, shoot:root ratio, specific leaf area, and leaf dry matter content). Data were analyzed using linear mixed effect models with tree biomass, earthworm treatment and species identity as well as the interaction between earthworm treatment and species identity as fixed effects, and EcoUnit specified as a random effect. The transformation applied on the data as well as the sample size are also presented.

| **Response** | **n** | **Transformation** | **Explanatory** | **Estimate** | **SE** | **t-value** |
| --- | --- | --- | --- | --- | --- | --- |
| Height | 480 | log10 | (Intercept) | 0.94 | 0.13 | 7 |
|  |  |  | tree_biomass | 0.00 | 0.00 | -2 |
|  |  |  | ew_presenceNoEW | -0.11 | 0.07 | -2 |
|  |  |  | species_idAM | 0.49 | 0.05 | 10 |
|  |  |  | species_idBC | 0.84 | 0.05 | 18 |
|  |  |  | species_idCC | 0.89 | 0.05 | 19 |
|  |  |  | ew_presenceNoEW:species_idAM | 0.12 | 0.07 | 2 |
|  |  |  | ew_presenceNoEW:species_idBC | 0.15 | 0.07 | 2 |
|  |  |  | ew_presenceNoEW:species_idCC | 0.01 | 0.07 | 0 |
| Shoot:root ratio | 96 | log10 | (Intercept) | 0.12 | 0.27 | 0 |
|  |  |  | tree_biomass | 0.00 | 0.00 | 0 |
|  |  |  | ew_presenceNoEW | -0.17 | 0.16 | -1 |
|  |  |  | species_idAM | 0.48 | 0.14 | 3 |
|  |  |  | species_idBC | 0.48 | 0.14 | 3 |
|  |  |  | species_idCC | 0.18 | 0.14 | 1 |
|  |  |  | ew_presenceNoEW:species_idAM | 0.20 | 0.20 | 1 |
|  |  |  | ew_presenceNoEW:species_idBC | 0.22 | 0.20 | 1 |
|  |  |  | ew_presenceNoEW:species_idCC | 0.25 | 0.20 | 1 |
| Specific leaf area | 240 | log10 | (Intercept) | 1.30 | 0.08 | 16 |
|  |  |  | tree_biomass | 0.00 | 0.00 | 2 |
|  |  |  | ew_presenceNoEW | 0.01 | 0.04 | 0 |
|  |  |  | species_idAM | -0.21 | 0.02 | -9 |
|  |  |  | species_idBC | 0.11 | 0.02 | 5 |
|  |  |  | species_idCC | 0.05 | 0.02 | 2 |
|  |  |  | ew_presenceNoEW:species_idAM | -0.01 | 0.03 | 0 |
|  |  |  | ew_presenceNoEW:species_idBC | -0.06 | 0.03 | -2 |
|  |  |  | ew_presenceNoEW:species_idCC | 0.04 | 0.03 | 1 |
| Leaf dry matter content | 240 | log10 | (Intercept) | 2.32 | 0.08 | 27 |
|  |  |  | tree_biomass | 0.00 | 0.00 | -2 |
|  |  |  | ew_presenceNoEW | 0.03 | 0.04 | 1 |
|  |  |  | species_idAM | -0.09 | 0.02 | -5 |
|  |  |  | species_idBC | 0.22 | 0.02 | 12 |
|  |  |  | species_idCC | 0.30 | 0.02 | 17 |
|  |  |  | ew_presenceNoEW:species_idAM | 0.01 | 0.02 | 0 |
|  |  |  | ew_presenceNoEW:species_idBC | 0.01 | 0.02 | 0 |
|  |  |  | ew_presenceNoEW:species_idCC | -0.04 | 0.02 | -2 |

**Table 4:** Model results for the effects of earthworms, species identity, and tree biomass on the number of ramets and proportion of flowers. Data were analyzed using linear mixed effect models with tree biomass, earthworm treatment and species identity as well as the interaction between earthworm treatment and species identity as fixed effects, and EcoUnit specified as a random effect. The transformation applied on the data as well as the sample size are also presented.

| **Response** | **n** | **Transformation** | **Explanatory** | **Estimate** | **SE** | **t-value** |
| --- | --- | --- | --- | --- | --- | --- |
| Number of ramets | 48 | log10 | (Intercept) | 0.05 | 0.11 | 0 |
|  |  |  | tree_biomass | 0.00 | 0.00 | 0 |
|  |  |  | ew_presenceNoEW | -0.01 | 0.08 | 0 |
|  |  |  | species_idAM | 0.18 | 0.08 | 2 |
|  |  |  | species_idBC | 0.81 | 0.08 | 10 |
|  |  |  | species_idCC | 0.88 | 0.08 | 11 |
|  |  |  | ew_presenceNoEW:species_idAM | 0.09 | 0.11 | 1 |
|  |  |  | ew_presenceNoEW:species_idBC | 0.09 | 0.11 | 1 |
|  |  |  | ew_presenceNoEW:species_idCC | 0.08 | 0.11 | 1 |
| Proportion of flowering individuals | 47 | logit | (Intercept) | -3.52 | 0.78 | -5 |
|  |  |  | tree_biomass | 0.00 | 0.00 | 0 |
|  |  |  | ew_presenceNoEW | 0.39 | 0.48 | 1 |
|  |  |  | species_idAM | 2.44 | 0.43 | 6 |
|  |  |  | species_idBC | 0.56 | 0.43 | 1 |
|  |  |  | species_idCC | 0.00 | 0.43 | 0 |
|  |  |  | ew_presenceNoEW:species_idAM | -0.86 | 0.60 | -1 |
|  |  |  | ew_presenceNoEW:species_idBC | -0.95 | 0.62 | -2 |
|  |  |  | ew_presenceNoEW:species_idCC | -0.39 | 0.60 | -1 |

**Table 5:** Model results for the effects of earthworms, species identity, and tree biomass on the nutrient content of the plant species (*i.e.* leaf carbon, nitrogen, C:N ratio, and leaf δ^15^N signature). Data were analyzed using linear mixed effect models with tree biomass, earthworm treatment and species identity as well as the interaction between earthworm treatment and species identity as fixed effects, and EcoUnit specified as a random effect. The transformation applied on the data as well as the sample size are also presented.

| **Response** | **n** | **Transformation** | **Explanatory** | **Estimate** | **SE** | **t-value** |
| --- | --- | --- | --- | --- | --- | --- |
| Leaf carbon content | 143 | none | (Intercept) | 43.97 | 0.87 | 51 |
|  |  |  | tree_biomass | 0.00 | 0.00 | 0 |
|  |  |  | ew_presenceNoEW | 0.12 | 0.49 | 0 |
|  |  |  | species_idAM | -2.16 | 0.40 | -5 |
|  |  |  | species_idBC | -0.52 | 0.40 | -1 |
|  |  |  | species_idCC | 0.25 | 0.40 | 1 |
|  |  |  | ew_presenceNoEW:species_idAM | 0.05 | 0.57 | 0 |
|  |  |  | ew_presenceNoEW:species_idBC | -0.23 | 0.57 | 0 |
|  |  |  | ew_presenceNoEW:species_idCC | -0.82 | 0.57 | -1 |
| Leaf nitrogen content | 143 | none | (Intercept) | 3.10 | 0.53 | 6 |
|  |  |  | tree_biomass | 0.00 | 0.00 | 2 |
|  |  |  | ew_presenceNoEW | 0.27 | 0.25 | 1 |
|  |  |  | species_idAM | -0.46 | 0.16 | -3 |
|  |  |  | species_idBC | -1.39 | 0.16 | -9 |
|  |  |  | species_idCC | -0.60 | 0.16 | -4 |
|  |  |  | ew_presenceNoEW:species_idAM | -0.30 | 0.23 | -1 |
|  |  |  | ew_presenceNoEW:species_idBC | 0.02 | 0.23 | 0 |
|  |  |  | ew_presenceNoEW:species_idCC | -0.32 | 0.23 | -1 |
| Leaf C:N ratio | 143 | log10 | (Intercept) | 1.16 | 0.07 | 17 |
|  |  |  | tree_biomass | 0.00 | 0.00 | -2 |
|  |  |  | ew_presenceNoEW | -0.03 | 0.03 | -1 |
|  |  |  | species_idAM | 0.03 | 0.02 | 2 |
|  |  |  | species_idBC | 0.19 | 0.02 | 9 |
|  |  |  | species_idCC | 0.08 | 0.02 | 4 |
|  |  |  | ew_presenceNoEW:species_idAM | 0.03 | 0.03 | 1 |
|  |  |  | ew_presenceNoEW:species_idBC | -0.02 | 0.03 | -1 |
|  |  |  | ew_presenceNoEW:species_idCC | 0.02 | 0.03 | 1 |
| Leaf d15N signature | 143 | none | (Intercept) | 7.40 | 1.13 | 7 |
|  |  |  | tree_biomass | 0.00 | 0.00 | 1 |
|  |  |  | ew_presenceNoEW | 0.09 | 0.61 | 0 |
|  |  |  | species_idAM | -0.68 | 0.47 | -1 |
|  |  |  | species_idBC | 0.31 | 0.47 | 1 |
|  |  |  | species_idCC | 0.83 | 0.47 | 2 |
|  |  |  | ew_presenceNoEW:species_idAM | 2.04 | 0.68 | 3 |
|  |  |  | ew_presenceNoEW:species_idBC | 0.89 | 0.68 | 1 |
|  |  |  | ew_presenceNoEW:species_idCC | 0.35 | 0.68 | 1 |

**Table 6:** Model results for the effects of earthworms, species identity, and tree biomass on root traits. Data were analyzed using linear mixed effect models with tree biomass, earthworm treatment and species identity as well as the interaction between earthworm treatment and species identity as fixed effects, and EcoUnit specified as a random effect. The transformation applied on the data as well as the sample size are also presented.

| **Response** | **n** | **Transformation** | **Explanatory** | **Estimate** | **SE** | **t-value** |
| --- | --- | --- | --- | --- | --- | --- |
| Root length | 96 | log10 | (Intercept) | 2.69 | 0.29 | 9 |
|  |  |  | tree_biomass | 0.00 | 0.00 | -1 |
|  |  |  | ew_presenceNoEW | -0.28 | 0.18 | -2 |
|  |  |  | species_idAM | 0.74 | 0.16 | 5 |
|  |  |  | species_idBC | 0.81 | 0.16 | 5 |
|  |  |  | species_idCC | 0.93 | 0.16 | 6 |
|  |  |  | ew_presenceNoEW:species_idAM | 0.43 | 0.22 | 2 |
|  |  |  | ew_presenceNoEW:species_idBC | 0.46 | 0.22 | 2 |
|  |  |  | ew_presenceNoEW:species_idCC | 0.72 | 0.22 | 3 |
| Root diameter | 96 | log10 | (Intercept) | -0.37 | 0.08 | -4 |
|  |  |  | tree_biomass | 0.00 | 0.00 | -1 |
|  |  |  | ew_presenceNoEW | 0.00 | 0.05 | 0 |
|  |  |  | species_idAM | -0.09 | 0.04 | -2 |
|  |  |  | species_idBC | -0.15 | 0.04 | -3 |
|  |  |  | species_idCC | -0.22 | 0.04 | -5 |
|  |  |  | ew_presenceNoEW:species_idAM | -0.03 | 0.06 | 0 |
|  |  |  | ew_presenceNoEW:species_idBC | 0.01 | 0.06 | 0 |
|  |  |  | ew_presenceNoEW:species_idCC | -0.01 | 0.06 | 0 |
| Root dry matter content | 96 | none | (Intercept) | 256.33 | 30.09 | 9 |
|  |  |  | tree_biomass | -0.09 | 0.04 | -2 |
|  |  |  | ew_presenceNoEW | 12.92 | 15.51 | 1 |
|  |  |  | species_idAM | -43.22 | 11.48 | -4 |
|  |  |  | species_idBC | -10.61 | 11.48 | -1 |
|  |  |  | species_idCC | -38.82 | 11.48 | -3 |
|  |  |  | ew_presenceNoEW:species_idAM | 6.96 | 16.24 | 0 |
|  |  |  | ew_presenceNoEW:species_idBC | -19.07 | 16.24 | -1 |
|  |  |  | ew_presenceNoEW:species_idCC | 2.35 | 16.24 | 0 |
| Root tissue density | 96 | none | (Intercept) | 0.44 | 0.05 | 8 |
|  |  |  | tree_biomass | 0.00 | 0.00 | -3 |
|  |  |  | ew_presenceNoEW | 0.00 | 0.03 | 0 |
|  |  |  | species_idAM | -0.07 | 0.02 | -3 |
|  |  |  | species_idBC | -0.01 | 0.02 | -1 |
|  |  |  | species_idCC | -0.05 | 0.02 | -2 |
|  |  |  | ew_presenceNoEW:species_idAM | 0.05 | 0.03 | 1 |
|  |  |  | ew_presenceNoEW:species_idBC | -0.02 | 0.03 | -1 |
|  |  |  | ew_presenceNoEW:species_idCC | 0.00 | 0.03 | 0 |
|  |  |  |  |  |  |  |
|  |  |  |  |  |  |  |
|  |  |  |  |  |  |  |
|  |  |  |  |  |  |  |
| Specific root length | 96 | log10 | (Intercept) | 1.15 | 0.18 | 6 |
|  |  |  | tree_biomass | 0.00 | 0.00 | 2 |
|  |  |  | ew_presenceNoEW | 0.01 | 0.11 | 0 |
|  |  |  | species_idAM | 0.31 | 0.10 | 3 |
|  |  |  | species_idBC | 0.32 | 0.10 | 3 |
|  |  |  | species_idCC | 0.52 | 0.10 | 5 |
|  |  |  | ew_presenceNoEW:species_idAM | -0.03 | 0.13 | 0 |
|  |  |  | ew_presenceNoEW:species_idBC | 0.00 | 0.13 | 0 |
|  |  |  | ew_presenceNoEW:species_idCC | 0.02 | 0.13 | 0 |
