# Supplementary material for: Do Invasive Earthworms Affect the Functional Traits of Native Plants?
Source: Front Plant Sci. 2021 Mar 16;12:627573. doi: 10.3389/fpls.2021.627573 (PMC8007962; doi:10.3389/fpls.2021.627573)
Supplement: Supplementary file 4 [file Data_Sheet_4.docx]

**Supplementary material 4**

**Do invasive earthworms affect the functional traits of native plants?**

**Lise Thouvenot^1,2*^, Olga Ferlian^1,2^, Remy Beugnon^1,2^, Tom Künne^1,2^, Alfred Lochner^1,2^, Madhav P. Thakur^1,2,3^, Manfred Türke^1,2^, and Nico Eisenhauer^1,2^**

^1^German Centre for Integrative Biodiversity Research (iDiv) Halle-Jena-Leipzig, Leipzig, Germany

^2^Institute of Biology, Leipzig University, Leipzig, Germany

^3^Terrestrial Ecology Group, University of Bern, Bern, Switzerland.

* **Correspondence:**

Lise Thouvenot

[lise.thouvenot@idiv.de](mailto:lise.thouvenot@idiv.de)

**Table 1:** Summary of Pearson’s correlation tests between the tree biomass and the functional traits of each plant species, performed when tree productivity significantly affected plant traits. Significant correlations are highlighted in bold.

|  | t value | Df | p-value | R² |
| --- | --- | --- | --- | --- |
| **Height** |  |  |  |  |
| *S. laeve* | -2.54 | 118 | **0.012** | -0.23 |
| *A. millefolium* | -1.11 | 118 | 0.269 | -0.10 |
| *B. ciliatus* | -1.52 | 118 | 0.131 | -0.14 |
| *C. canadensis* | -3.00 | 118 | **0.003** | -0.26 |
| **Root dry matter content** |  |  |  |  |
| *S. laeve* | -2.10 | 22 | **0.047** | -0.41 |
| *A. millefolium* | -1.82 | 22 | 0.082 | -0.36 |
| *B. ciliatus* | -1.88 | 22 | 0.074 | -0.37 |
| *C. canadensis* | -2.03 | 22 | 0.056 | -0.40 |
| **Root tissue density** |  |  |  |  |
| *S. laeve* | -3.12 | 22 | **0.005** | -0.55 |
| *A. millefolium* | -2.20 | 22 | **0.038** | -0.42 |
| *B. ciliatus* | -0.87 | 22 | 0.39 | -0.18 |
| *C. canadensis* | -3.03 | 22 | **0.006** | -0.54 |
