# Supplementary material for: Do Invasive Earthworms Affect the Functional Traits of Native Plants?
Source: Front Plant Sci. 2021 Mar 16;12:627573. doi: 10.3389/fpls.2021.627573 (PMC8007962; doi:10.3389/fpls.2021.627573)
Supplement: Supplementary file 5 [file Data_Sheet_5.docx]

**Supplementary material 5**

**Do invasive earthworms affect the functional traits of native plants?**

**Lise Thouvenot^1,2*^, Olga Ferlian^1,2^, Remy Beugnon^1,2^, Tom Künne^1,2^, Alfred Lochner^1,2^, Madhav P. Thakur^1,2,3^, Manfred Türke^1,2^, and Nico Eisenhauer^1,2^**

^1^German Centre for Integrative Biodiversity Research (iDiv) Halle-Jena-Leipzig, Leipzig, Germany

^2^Institute of Biology, Leipzig University, Leipzig, Germany

^3^Terrestrial Ecology Group, University of Bern, Bern, Switzerland.

* **Correspondence:**

Lise Thouvenot

[lise.thouvenot@idiv.de](mailto:lise.thouvenot@idiv.de)

|  | **Height** | | | | **Specific leaf area** | | | | **Leaf dry matter content** | | | | **δ^15^N in leaves** | | | | **Root length** | | | |
| --- | --- | --- | --- | --- | --- | --- | --- | --- | --- | --- | --- | --- | --- | --- | --- | --- | --- | --- | --- | --- |
| contrast | estimate | Df | t ratio | p-value | estimate | Df | t ratio | p-value | estimate | Df | t ratio | p-value | estimate | Df | t ratio | p-value | estimate | Df | t ratio | p-value |
| **Control** |  |  |  |  |  |  |  |  |  |  |  |  |  |  |  |  |  |  |  |  |
| *S. laeve*-  *A. millefolium* | -0.61 | 462.00 | -12.98 | **<0.001** | 0.22 | 222.00 | 9.34 | **<0.001** | 0.08 | 222.00 | 4.40 | **<0.001** | -1.36 | 125.17 | -2.82 | **0.033** | -1.17 | 78.00 | -7.36 | **<0.001** |
| *S. laeve*-  *B. ciliatus* | -0.99 | 462.00 | -21.15 | **<0.001** | -0.06 | 222.00 | -2.38 | **0.037** | -0.23 | 222.00 | -12.95 | **<0.001** | -1.20 | 125.17 | -2.49 | 0.071 | -1.28 | 78.00 | -8.04 | **<0.001** |
| *S. laeve*-  *C. canadensis* | -0.89 | 462.00 | -19.06 | **<0.001** | -0.09 | 222.00 | -3.92 | **<0.001** | -0.26 | 222.00 | -14.80 | **<0.001** | -1.18 | 125.17 | -2.45 | 0.071 | -1.65 | 78.00 | -10.43 | **<0.001** |
| *A. millefolium - B. ciliatus* | -0.38 | 462.00 | -8.18 | **<0.001** | -0.27 | 222.00 | -11.71 | **<0.001** | -0.30 | 222.00 | -17.35 | **<0.001** | 0.16 | 125.00 | 0.34 | 1.000 | -0.11 | 78.00 | -0.68 | 0.498 |
| *A. millefolium - C. canadensis* | -0.28 | 462.00 | -6.08 | **<0.001** | -0.31 | 222.00 | -13.26 | **<0.001** | -0.34 | 222.00 | -19.20 | **<0.001** | 0.18 | 125.00 | 0.38 | 1.000 | -0.49 | 78.00 | -3.07 | **0.009** |
| *B. ciliatus -*  *C. canadensis* | 0.10 | 462.00 | 2.10 | **0.037** | -0.04 | 222.00 | -1.54 | 0.124 | -0.03 | 222.00 | -1.85 | 0.066 | 0.02 | 125.00 | 0.04 | 1.000 | -0.38 | 78.00 | -2.39 | **0.039** |
| **Earthworm** |  |  |  |  |  |  |  |  |  |  |  |  |  |  |  |  |  |  |  |  |
| *S. laeve*-  *A. millefolium* | -0.49 | 462.00 | -10.45 | **<0.001** | 0.21 | 222.00 | 9.02 | **<0.001** | 0.09 | 222.00 | 4.99 | **<0.001** | 0.68 | 125.00 | 1.44 | 0.457 | -0.74 | 78.00 | -4.68 | **<0.001** |
| *S. laeve*-  *B. ciliatus* | -0.84 | 462.00 | -17.88 | **<0.001** | -0.11 | 222.00 | -4.76 | **<0.001** | -0.22 | 222.00 | -12.39 | **<0.001** | -0.31 | 125.00 | -0.65 | 0.546 | -0.81 | 78.00 | -5.11 | **<0.001** |
| *S. laeve*-  *C. canadensis* | -0.89 | 462.00 | -18.92 | **<0.001** | -0.05 | 222.00 | -2.25 | **0.026** | -0.30 | 222.00 | -17.25 | **<0.001** | -0.83 | 125.00 | -1.75 | 0.329 | -0.93 | 78.00 | -5.86 | **<0.001** |
| *A. millefolium - B. ciliatus* | -0.35 | 462.00 | -7.43 | **<0.001** | -0.32 | 222.00 | -13.78 | **<0.001** | -0.31 | 222.00 | -17.37 | **<0.001** | -0.99 | 125.00 | -2.09 | 0.193 | -0.07 | 78.00 | -0.44 | 0.913 |
| *A. millefolium - C. canadensis* | -0.40 | 462.00 | -8.47 | **<0.001** | -0.26 | 222.00 | -11.27 | **<0.001** | -0.39 | 222.00 | -22.24 | **<0.001** | -1.51 | 125.00 | -3.19 | **0.011** | -0.19 | 78.00 | -1.18 | 0.720 |
| *B. ciliatus -*  *C. canadensis* | -0.05 | 462.00 | -1.04 | 0.300 | 0.06 | 222.00 | 2.51 | **0.026** | -0.09 | 222.00 | -4.86 | **<0.001** | -0.52 | 125.00 | -1.10 | 0.546 | -0.12 | 78.00 | -0.75 | 0.913 |

**Table 1:** Results of post-hoc comparisons (with the Holm correction applied to adjust the p-values) by earthworm treatment that were applied in case of a significant interaction effect of earthworm presence and species identity (*i.e.* for height, the specific leaf area, leaf dry matter content, δ^15^N signatures in leaves, and root length). Plant species were compared within each earthworm treatment. Significant effects are highlighted in bold.

**Table 2:** Results of post-hoc comparisons (with the Holm correction applied to adjust the p-values) by plant species that were applied in case of a significant interaction effect of earthworm presence and plant species identity (i.e. for the height, the specific leaf area, the leaf dry matter content, the δ^15^N signature in leaves, and the root length). Earthworm treatments were compared per plant species. Significant effects are highlighted in bold.

|  | **contrast** | **estimate** | **Df** | **t ratio** | **p-value** |
| --- | --- | --- | --- | --- | --- |
| **Height** |  |  |  |  |  |
| *S. laeve* | Earthworm-Control | 0.11 | 23.18 | 1.63 | 0.116 |
| *A. millefolium* | Earthworm-Control | -0.01 | 23.18 | -0.16 | 0.872 |
| *B. ciliatus* | Earthworm-Control | -0.05 | 23.18 | -0.69 | 0.494 |
| *C. canadensis* | Earthworm-Control | 0.10 | 23.18 | 1.53 | 0.139 |
| **Specific leaf area** |  |  |  |  |  |
| *S. laeve* | Earthworm-Control | -0.01 | 17.10 | -0.14 | 0.892 |
| *A. millefolium* | Earthworm-Control | 0.01 | 17.10 | 0.05 | 0.959 |
| *B. ciliatus* | Earthworm-Control | 0.05 | 17.10 | 1.31 | 0.208 |
| *C. canadensis* | Earthworm-Control | -0.04 | 17.10 | -1.15 | 0.265 |
| **Leaf dry matter content** |  |  |  |  |  |
| *S. laeve* | Earthworm-Control | -0.03 | 12.90 | -0.71 | 0.493 |
| *A. millefolium* | Earthworm-Control | -0.04 | 12.90 | -0.98 | 0.344 |
| *B. ciliatus* | Earthworm-Control | -0.04 | 12.90 | -0.97 | 0.350 |
| *C. canadensis* | Earthworm-Control | 0.02 | 12.90 | 0.44 | 0.663 |
| **δ^15^N in leaves** |  |  |  |  |  |
| *S. laeve* | Earthworm-Control | -0.09 | 30.50 | -0.15 | 0.881 |
| *A. millefolium* | Earthworm-Control | -2.14 | 29.50 | -3.54 | **0.001** |
| *B. ciliatus* | Earthworm-Control | -0.98 | 29.50 | -1.63 | 0.115 |
| *C. canadensis* | Earthworm-Control | -0.44 | 29.50 | -0.73 | 0.471 |
| **Root length** |  |  |  |  |  |
| *S. laeve* | Earthworm-Control | 0.28 | 45.42 | 1.60 | 0.116 |
| *A. millefolium* | Earthworm-Control | -0.14 | 45.42 | -0.81 | 0.423 |
| *B. ciliatus* | Earthworm-Control | -0.18 | 45.42 | -1.03 | 0.309 |
| *C. canadensis* | Earthworm-Control | -0.44 | 45.42 | -2.50 | **0.016** |
