# Supplementary material for: Do Invasive Earthworms Affect the Functional Traits of Native Plants?
Source: Front Plant Sci. 2021 Mar 16;12:627573. doi: 10.3389/fpls.2021.627573 (PMC8007962; doi:10.3389/fpls.2021.627573)
Supplement: Supplementary file 6 [file Data_Sheet_6.docx]

**Supplementary material 6**

**Do invasive earthworms affect the functional traits of native plants?**

**Lise Thouvenot^1,2*^, Olga Ferlian^1,2^, Remy Beugnon^1,2^, Tom Künne^1,2^, Alfred Lochner^1,2^, Madhav P. Thakur^1,2,3^, Manfred Türke^1,2^, and Nico Eisenhauer^1,2^**

^1^German Centre for Integrative Biodiversity Research (iDiv) Halle-Jena-Leipzig, Leipzig, Germany

^2^Institute of Biology, Leipzig University, Leipzig, Germany

^3^Terrestrial Ecology Group, University of Bern, Bern, Switzerland.

* **Correspondence:**

Lise Thouvenot

[lise.thouvenot@idiv.de](mailto:lise.thouvenot@idiv.de)

**Table 1:** Spearman’s correlations between plant functional traits (*i.e.* mean per EcoUnit) of *Calamagrostis canadensis.* Correlation coefficients and p-values (in brackets) above the diagonal correspond to the ones in the presence of earthworms, while those below the diagonal are those from the species *Calamagrostis canadensis* in the absence of earthworms. Abbreviations are: SLA=specific leaf area; LDMC=leaf dry matter content; RL=root length; RD=root diameter; RDMC=root dry matter content; RTD=root tissue density; SRL=specific root length; LCC=leaf carbon content; LNC=leaf nitrogen content; PFI=proportion of flowering individuals. Number of observations per treatment: 6. Significant correlations are highlighted in bold.

| ***Calamagrostis canadensis*** | | **Earthworm presence** | | | | | | | | | | | | | | |
| --- | --- | --- | --- | --- | --- | --- | --- | --- | --- | --- | --- | --- | --- | --- | --- | --- |
|  |  | Height | S:R ratio | SLA | LDMC | RL | RD | RDMC | RTD | SRL | LCC | LNC | C:N ratio | Leaf 15N | Ramets | PFI |
| **Earthworm absence** | Height |  | -0.2 (0.704) | -0.086 (0.872) | 0.371 (0.468) | 0.657 (0.156) | 0.29 (0.577) | 0.6 (0.208) | **0.886 (0.019)** | -0.429 (0.397) | 0.143 (0.787) | -0.543 (0.266) | 0.429 (0.397) | 0.771 (0.072) | 0.771 (0.072) | NA |
|  | S:R ratio | 0.6 (0.208) |  | 0.429 (0.397) | -0.486 (0.329) | 0.6 (0.208) | -0.725 (0.103) | -0.486 (0.329) | -0.371 (0.468) | **0.829 (0.042)** | 0.086 (0.872) | 0.371 (0.468) | -0.429 (0.397) | -0.543 (0.266) | 0.086 (0.872) | NA |
|  | SLA | 0.143 (0.787) | 0.543 (0.266) |  | **-0.943 (0.005)** | 0.257 (0.623) | 0.29 (0.577) | -0.657 (0.156) | -0.486 (0.329) | 0.029 (0.957) | 0.771 (0.072) | -0.086 (0.872) | 0.143 (0.787) | -0.029 (0.957) | 0.486 (0.329) | NA |
|  | LDMC | -0.143 (0.787) | -0.543 (0.266) | **-1 (0)** |  | -0.086 (0.872) | -0.145 (0.784) | **0.829 (0.042)** | 0.714 (0.111) | -0.143 (0.787) | -0.6 (0.208) | -0.029 (0.957) | -0.086 (0.872) | 0.2 (0.704) | -0.257 (0.623) | NA |
|  | RL | -0.086 (0.872) | -0.371 (0.468) | 0.429 (0.397) | -0.429 (0.397) |  | -0.319 (0.538) | 0.086 (0.872) | 0.429 (0.397) | 0.257 (0.623) | 0.143 (0.787) | -0.2 (0.704) | 0.086 (0.872) | 0.257 (0.623) | 0.714 (0.111) | NA |
|  | RD | -0.029 (0.957) | -0.143 (0.787) | 0.657 (0.156) | -0.657 (0.156) | **0.943 (0.005)** |  | 0.145 (0.784) | 0.174 (0.742) | **-0.899 (0.015)** | 0.464 (0.354) | -0.58 (0.228) | 0.638 (0.173) | 0.638 (0.173) | 0.406 (0.425) | NA |
|  | RDMC | 0.314 (0.544) | -0.314 (0.544) | -0.086 (0.872) | 0.086 (0.872) | 0.486 (0.329) | 0.257 (0.623) |  | **0.886 (0.019)** | -0.371 (0.468) | -0.429 (0.397) | -0.429 (0.397) | 0.2 (0.704) | 0.371 (0.468) | 0.143 (0.787) | NA |
|  | RTD | 0.257 (0.623) | -0.543 (0.266) | -0.257 (0.623) | 0.257 (0.623) | 0.543 (0.266) | 0.429 (0.397) | 0.486 (0.329) |  | -0.429 (0.397) | -0.257 (0.623) | -0.543 (0.266) | 0.371 (0.468) | 0.657 (0.156) | 0.486 (0.329) | NA |
|  | SRL | -0.257 (0.623) | 0.543 (0.266) | 0.257 (0.623) | -0.257 (0.623) | -0.543 (0.266) | -0.429 (0.397) | -0.486 (0.329) | **-1 (0)** |  | -0.086 (0.872) | 0.771 (0.072) | **-0.829 (0.042)** | **-0.829 (0.042)** | -0.429 (0.397) | NA |
|  | LCC | 0.086 (0.872) | -0.143 (0.787) | 0.543 (0.266) | -0.543 (0.266) | **0.943 (0.005)** | **0.886 (0.019)** | 0.6 (0.208) | 0.371 (0.468) | -0.371 (0.468) |  | 0.143 (0.787) | -0.086 (0.872) | 0.086 (0.872) | 0.371 (0.468) | NA |
|  | LNC | -0.029 (0.957) | 0.6 (0.208) | 0.086 (0.872) | -0.086 (0.872) | -0.657 (0.156) | -0.6 (0.208) | -0.314 (0.544) | **-0.943 (0.005)** | **0.943 (0.005)** | -0.429 (0.397) |  | **-0.943 (0.005)** | -0.771 (0.072) | -0.714 (0.111) | NA |
|  | C:N ratio | 0.086 (0.872) | -0.486 (0.329) | 0.086 (0.872) | -0.086 (0.872) | **0.829 (0.042)** | 0.771 (0.072) | 0.371 (0.468) | **0.886 (0.019)** | **-0.886 (0.019)** | 0.657 (0.156) | **-0.943 (0.005)** |  | **0.829 (0.042)** | 0.657 (0.156) | NA |
|  | Leaf 15N | 0.086 (0.872) | 0.2 (0.704) | -0.143 (0.787) | 0.143 (0.787) | -0.314 (0.544) | -0.143 (0.787) | -0.771 (0.072) | 0.086 (0.872) | -0.086 (0.872) | -0.486 (0.329) | -0.143 (0.787) | 0.086 (0.872) |  | 0.714 (0.111) | NA |
|  | Ramets | 0.314 (0.544) | -0.029 (0.957) | **-0.829 (0.042)** | **0.829 (0.042)** | -0.657 (0.156) | **-0.829 (0.042)** | 0.143 (0.787) | 0.029 (0.957) | -0.029 (0.957) | -0.6 (0.208) | 0.257 (0.623) | -0.371 (0.468) | 0.086 (0.872) |  | NA |
|  | PFI | NA | NA | NA | NA | NA | NA | NA | NA | NA | NA | NA | NA | NA | NA |  |

**Table 2:** Spearman’s correlations between plant functional traits (*i.e.* mean per EcoUnit) of *Bromus ciliatus*. Correlation coefficients and p-values (in brackets) above the diagonal correspond to the ones in the presence of earthworms, while those below the diagonal are those from the species *Bromus ciliatus* in the absence of earthworms. Abbreviations are: SLA=specific leaf area; LDMC=leaf dry matter content; RL=root length; RD=root diameter; RDMC=root dry matter content; RTD=root tissue density; SRL=specific root length; LCC=leaf carbon content; LNC=leaf nitrogen content; PFI=proportion of flowering individuals. Number of observations per treatment: 6. Significant correlations are highlighted in bold.

| ***Bromus ciliatus*** | | **Earthworm presence** | | | | | | | | | | | | | | |
| --- | --- | --- | --- | --- | --- | --- | --- | --- | --- | --- | --- | --- | --- | --- | --- | --- |
|  |  | Height | S:R ratio | SLA | LDMC | RL | RD | RDMC | RTD | SRL | LCC | LNC | C:N ratio | Leaf 15N | Ramets | PFI |
| **Earthworm absence** | Height |  | 0.714 (0.111) | -0.086 (0.872) | 0.371 (0.468) | -0.143 (0.787) | 0.522 (0.288) | **0.886 (0.019)** | **0.829 (0.042)** | -0.657 (0.156) | -0.429 (0.397) | **-0.943 (0.005)** | **0.943 (0.005)** | -0.314 (0.544) | -0.257 (0.623) | 0.621 (0.188) |
|  | S:R ratio | 0.771 (0.072) |  | 0.429 (0.397) | -0.257 (0.623) | -0.029 (0.957) | **0.841 (0.036)** | **0.829 (0.042)** | **0.886 (0.019)** | **-0.886 (0.019)** | 0.314 (0.544) | -0.543 (0.266) | 0.543 (0.266) | -0.143 (0.787) | 0.086 (0.872) | 0.414 (0.414) |
|  | SLA | -0.371 (0.468) | -0.6 (0.208) |  | **-0.829 (0.042)** | -0.429 (0.397) | 0.696 (0.125) | 0.257 (0.623) | 0.086 (0.872) | -0.6 (0.208) | 0.657 (0.156) | 0.257 (0.623) | -0.257 (0.623) | -0.143 (0.787) | -0.314 (0.544) | -0.207 (0.694) |
|  | LDMC | 0.429 (0.397) | 0.657 (0.156) | -0.771 (0.072) |  | 0.086 (0.872) | -0.377 (0.461) | 0.029 (0.957) | 0.143 (0.787) | 0.257 (0.623) | **-0.886 (0.019)** | -0.429 (0.397) | 0.429 (0.397) | 0.257 (0.623) | -0.143 (0.787) | 0.621 (0.188) |
|  | RL | 0.771 (0.072) | 0.314 (0.544) | -0.257 (0.623) | 0.086 (0.872) |  | -0.116 (0.827) | -0.029 (0.957) | 0.2 (0.704) | 0.143 (0.787) | 0.143 (0.787) | -0.029 (0.957) | 0.029 (0.957) | 0.371 (0.468) | 0.771 (0.072) | -0.207 (0.694) |
|  | RD | 0.58 (0.228) | 0.754 (0.084) | -0.29 (0.577) | 0.754 (0.084) | 0 (1) |  | **0.812 (0.05)** | 0.754 (0.084) | **-0.986 (0)** | 0.319 (0.538) | -0.348 (0.499) | 0.348 (0.499) | 0.029 (0.957) | -0.232 (0.658) | 0.315 (0.543) |
|  | RDMC | 0.771 (0.072) | 0.657 (0.156) | -0.086 (0.872) | 0.543 (0.266) | 0.314 (0.544) | **0.899 (0.015)** |  | **0.886 (0.019)** | **-0.886 (0.019)** | -0.143 (0.787) | **-0.829 (0.042)** | **0.829 (0.042)** | -0.257 (0.623) | -0.257 (0.623) | 0.414 (0.414) |
|  | RTD | 0.771 (0.072) | 0.657 (0.156) | -0.086 (0.872) | 0.543 (0.266) | 0.314 (0.544) | **0.899 (0.015)** | **1 (0)** |  | **-0.829 (0.042)** | -0.029 (0.957) | -0.714 (0.111) | 0.714 (0.111) | 0.086 (0.872) | 0.086 (0.872) | 0.621 (0.188) |
|  | SRL | -0.657 (0.156) | -0.771 (0.072) | 0.2 (0.704) | -0.657 (0.156) | -0.086 (0.872) | **-0.986 (0)** | **-0.943 (0.005)** | **-0.943 (0.005)** |  | -0.2 (0.704) | 0.486 (0.329) | -0.486 (0.329) | 0.029 (0.957) | 0.257 (0.623) | -0.414 (0.414) |
|  | LCC | 0.6 (0.208) | 0.714 (0.111) | **-0.943 (0.005)** | **0.829 (0.042)** | 0.371 (0.468) | 0.493 (0.321) | 0.371 (0.468) | 0.371 (0.468) | -0.429 (0.397) |  | 0.543 (0.266) | -0.543 (0.266) | 0.086 (0.872) | 0.486 (0.329) | -0.414 (0.414) |
|  | LNC | 0.486 (0.329) | 0.6 (0.208) | **-0.886 (0.019)** | **0.943 (0.005)** | 0.257 (0.623) | 0.58 (0.228) | 0.429 (0.397) | 0.429 (0.397) | -0.486 (0.329) | **0.943 (0.005)** |  | **-1 (0)** | 0.429 (0.397) | 0.2 (0.704) | -0.414 (0.414) |
|  | C:N ratio | -0.486 (0.329) | -0.6 (0.208) | **0.886 (0.019)** | **-0.943 (0.005)** | -0.257 (0.623) | -0.58 (0.228) | -0.429 (0.397) | -0.429 (0.397) | 0.486 (0.329) | **-0.943 (0.005)** | **-1 (0)** |  | -0.429 (0.397) | -0.2 (0.704) | 0.414 (0.414) |
|  | Leaf 15N | 0.2 (0.704) | 0.314 (0.544) | -0.143 (0.787) | 0.714 (0.111) | -0.257 (0.623) | **0.841 (0.036)** | 0.714 (0.111) | 0.714 (0.111) | -0.771 (0.072) | 0.314 (0.544) | 0.543 (0.266) | -0.543 (0.266) |  | 0.257 (0.623) | 0.414 (0.414) |
|  | Ramets | 0.657 (0.156) | 0.543 (0.266) | -0.714 (0.111) | 0.657 (0.156) | 0.429 (0.397) | 0.464 (0.354) | 0.486 (0.329) | 0.486 (0.329) | -0.429 (0.397) | **0.886 (0.019)** | **0.829 (0.042)** | **-0.829 (0.042)** | 0.371 (0.468) |  | -0.207 (0.694) |
|  | PFI | NA | NA | NA | NA | NA | NA | NA | NA | NA | NA | NA | NA | NA | NA |  |

**Table 3:** Spearman’s correlations between plant functional traits (*i.e.* mean per EcoUnit) of *Achillea millefolium* Correlation coefficients and p-values (in brackets) above the diagonal correspond to the ones in the presence of earthworms, while those below the diagonal are those from the species *Achillea millefolium* in the absence of earthworms. Abbreviations are: SLA=specific leaf area; LDMC=leaf dry matter content; RL=root length; RD=root diameter; RDMC=root dry matter content; RTD=root tissue density; SRL=specific root length; LCC=leaf carbon content; LNC=leaf nitrogen content; PFI=proportion of flowering individuals. Number of observations per treatment: 6. Significant correlations are highlighted in bold.

| ***Achillea millefolium*** | | **Earthworm presence** | | | | | | | | | | | | | | |
| --- | --- | --- | --- | --- | --- | --- | --- | --- | --- | --- | --- | --- | --- | --- | --- | --- |
|  |  | Height | S:R ratio | SLA | LDMC | RL | RD | RDMC | RTD | SRL | LCC | LNC | C:N ratio | Leaf 15N | Ramets | PFI |
| **Earthworm absence** | Height |  | 0.771 (0.072) | -0.429 (0.397) | 0.143 (0.787) | -0.6 (0.208) | 0.486 (0.329) | 0.086 (0.872) | -0.086 (0.872) | -0.257 (0.623) | 0.143 (0.787) | -0.6 (0.208) | 0.6 (0.208) | 0.371 (0.468) | 0.174 (0.742) | **0.812 (0.05)** |
|  | S:R ratio | **-0.943 (0.005)** |  | -0.771 (0.072) | 0.6 (0.208) | **-0.829 (0.042)** | 0.429 (0.397) | -0.371 (0.468) | -0.6 (0.208) | 0.257 (0.623) | -0.429 (0.397) | -0.143 (0.787) | 0.143 (0.787) | 0.029 (0.957) | 0.609 (0.2) | **0.928 (0.008)** |
|  | SLA | 0.2 (0.704) | -0.086 (0.872) |  | **-0.943 (0.005)** | 0.486 (0.329) | -0.2 (0.704) | 0.257 (0.623) | 0.486 (0.329) | -0.371 (0.468) | 0.543 (0.266) | 0.029 (0.957) | -0.029 (0.957) | 0.429 (0.397) | -0.609 (0.2) | **-0.812 (0.05)** |
|  | LDMC | 0.143 (0.787) | -0.257 (0.623) | **-0.886 (0.019)** |  | -0.429 (0.397) | -0.029 (0.957) | -0.429 (0.397) | -0.6 (0.208) | 0.6 (0.208) | -0.714 (0.111) | 0.257 (0.623) | -0.257 (0.623) | -0.486 (0.329) | 0.522 (0.288) | 0.638 (0.173) |
|  | RL | -0.771 (0.072) | **0.829 (0.042)** | 0.257 (0.623) | -0.657 (0.156) |  | -0.143 (0.787) | 0.714 (0.111) | **0.829 (0.042)** | -0.543 (0.266) | 0.6 (0.208) | -0.2 (0.704) | 0.2 (0.704) | -0.429 (0.397) | -0.232 (0.658) | -0.754 (0.084) |
|  | RD | -0.145 (0.784) | 0.058 (0.913) | 0.058 (0.913) | -0.377 (0.461) | 0.493 (0.321) |  | 0.429 (0.397) | 0.143 (0.787) | -0.543 (0.266) | 0.029 (0.957) | -0.714 (0.111) | 0.714 (0.111) | 0.086 (0.872) | 0.319 (0.538) | 0.493 (0.321) |
|  | RDMC | 0.486 (0.329) | -0.543 (0.266) | 0.486 (0.329) | -0.143 (0.787) | -0.429 (0.397) | -0.406 (0.425) |  | **0.943 (0.005)** | **-0.943 (0.005)** | 0.771 (0.072) | **-0.829 (0.042)** | **0.829 (0.042)** | -0.143 (0.787) | -0.174 (0.742) | -0.203 (0.7) |
|  | RTD | 0.143 (0.787) | -0.086 (0.872) | 0.257 (0.623) | -0.029 (0.957) | -0.2 (0.704) | -0.696 (0.125) | 0.771 (0.072) |  | **-0.886 (0.019)** | **0.886 (0.019)** | -0.657 (0.156) | 0.657 (0.156) | -0.086 (0.872) | -0.348 (0.499) | -0.464 (0.354) |
|  | SRL | -0.2 (0.704) | 0.086 (0.872) | -0.657 (0.156) | 0.771 (0.072) | -0.429 (0.397) | -0.493 (0.321) | -0.257 (0.623) | -0.143 (0.787) |  | **-0.829 (0.042)** | **0.886 (0.019)** | **-0.886 (0.019)** | -0.086 (0.872) | 0.174 (0.742) | 0.145 (0.784) |
|  | LCC | -0.371 (0.468) | 0.257 (0.623) | -0.143 (0.787) | 0.029 (0.957) | 0.257 (0.623) | -0.058 (0.913) | 0.371 (0.468) | 0.6 (0.208) | -0.2 (0.704) |  | -0.6 (0.208) | 0.6 (0.208) | 0.086 (0.872) | -0.29 (0.577) | -0.406 (0.425) |
|  | LNC | -0.714 (0.111) | 0.543 (0.266) | -0.714 (0.111) | 0.543 (0.266) | 0.143 (0.787) | -0.087 (0.87) | -0.486 (0.329) | -0.257 (0.623) | 0.771 (0.072) | 0.2 (0.704) |  | **-1 (0)** | -0.143 (0.787) | 0.058 (0.913) | -0.319 (0.538) |
|  | C:N ratio | 0.714 (0.111) | -0.543 (0.266) | 0.714 (0.111) | -0.543 (0.266) | -0.143 (0.787) | 0.087 (0.87) | 0.486 (0.329) | 0.257 (0.623) | -0.771 (0.072) | -0.2 (0.704) | **-1 (0)** |  | 0.143 (0.787) | -0.058 (0.913) | 0.319 (0.538) |
|  | Leaf 15N | 0.371 (0.468) | -0.143 (0.787) | -0.314 (0.544) | 0.314 (0.544) | -0.257 (0.623) | -0.116 (0.827) | -0.486 (0.329) | -0.257 (0.623) | 0.086 (0.872) | -0.543 (0.266) | -0.2 (0.704) | 0.2 (0.704) |  | -0.667 (0.148) | 0.116 (0.827) |
|  | Ramets | -0.371 (0.468) | 0.429 (0.397) | 0.6 (0.208) | **-0.886 (0.019)** | **0.829 (0.042)** | 0.696 (0.125) | -0.257 (0.623) | -0.371 (0.468) | -0.657 (0.156) | -0.086 (0.872) | -0.257 (0.623) | 0.257 (0.623) | -0.257 (0.623) |  | 0.382 (0.454) |
|  | PFI | 0.348 (0.499) | -0.058 (0.913) | 0.551 (0.257) | -0.58 (0.228) | 0.232 (0.658) | 0.103 (0.846) | 0.029 (0.957) | 0.145 (0.784) | -0.754 (0.084) | -0.203 (0.7) | **-0.812 (0.05)** | **0.812 (0.05)** | 0.493 (0.321) | 0.406 (0.425) |  |

**Table 4:** Spearman’s correlations between plant functional traits (*i.e.* mean per EcoUnit) of *Symphyotrichum laeve*. Correlation coefficients and p-values (in brackets) above the diagonal correspond to the ones in the presence of earthworms, while those below the diagonal are those from the species *Aster laevis* in the absence of earthworms. Abbreviations are: SLA=specific leaf area; LDMC=leaf dry matter content; RL=root length; RD=root diameter; RDMC=root dry matter content; RTD=root tissue density; SRL=specific root length; LCC=leaf carbon content; LNC=leaf nitrogen content; PFI= proportion of flowering individuals. Number of observations per treatment: 6. Significant correlations are highlighted in bold.

| ***Symphyotrichum laeve*** | | **Earthworm presence** | | | | | | | | | | | | | | |
| --- | --- | --- | --- | --- | --- | --- | --- | --- | --- | --- | --- | --- | --- | --- | --- | --- |
|  |  | Height | S:R ratio | SLA | LDMC | RL | RD | RDMC | RTD | SRL | LCC | LNC | C:N ratio | Leaf 15N | Ramets | PFI |
| **Earthworm absence** | Height |  | 0.714 (0.111) | -0.371 (0.468) | 0.257 (0.623) | 0.486 (0.329) | **0.943 (0.005)** | 0.6 (0.208) | 0.486 (0.329) | -0.771 (0.072) | **-0.943 (0.005)** | 0.6 (0.208) | -0.771 (0.072) | 0.771 (0.072) | **-0.926 (0.008)** | NA |
|  | S:R ratio | 0.486 (0.329) |  | -0.6 (0.208) | 0.657 (0.156) | 0.257 (0.623) | 0.6 (0.208) | 0.486 (0.329) | 0.543 (0.266) | -0.6 (0.208) | -0.657 (0.156) | 0.543 (0.266) | -0.6 (0.208) | 0.543 (0.266) | -0.772 (0.072) | NA |
|  | SLA | 0.029 (0.957) | 0.2 (0.704) |  | **-0.943 (0.005)** | 0.143 (0.787) | -0.314 (0.544) | -0.086 (0.872) | -0.029 (0.957) | 0.2 (0.704) | 0.143 (0.787) | -0.771 (0.072) | 0.657 (0.156) | -0.6 (0.208) | 0.617 (0.192) | NA |
|  | LDMC | -0.429 (0.397) | -0.086 (0.872) | -0.257 (0.623) |  | -0.086 (0.872) | 0.143 (0.787) | 0.029 (0.957) | 0.086 (0.872) | -0.029 (0.957) | -0.029 (0.957) | 0.657 (0.156) | -0.486 (0.329) | 0.371 (0.468) | -0.463 (0.355) | NA |
|  | RL | 0.257 (0.623) | 0.771 (0.072) | 0.543 (0.266) | -0.029 (0.957) |  | 0.543 (0.266) | 0.771 (0.072) | **0.829 (0.042)** | -0.314 (0.544) | -0.371 (0.468) | -0.257 (0.623) | 0.143 (0.787) | 0.257 (0.623) | -0.309 (0.552) | NA |
|  | RD | **0.829 (0.042)** | 0.657 (0.156) | -0.143 (0.787) | -0.6 (0.208) | 0.314 (0.544) |  | 0.771 (0.072) | 0.6 (0.208) | **-0.886 (0.019)** | **-0.886 (0.019)** | 0.429 (0.397) | -0.657 (0.156) | **0.886 (0.019)** | **-0.926 (0.008)** | NA |
|  | RDMC | -0.543 (0.266) | -0.029 (0.957) | -0.143 (0.787) | -0.257 (0.623) | 0.086 (0.872) | -0.029 (0.957) |  | **0.943 (0.005)** | -0.771 (0.072) | -0.543 (0.266) | -0.143 (0.787) | -0.086 (0.872) | 0.657 (0.156) | -0.617 (0.192) | NA |
|  | RTD | -0.543 (0.266) | -0.029 (0.957) | -0.143 (0.787) | -0.257 (0.623) | 0.086 (0.872) | -0.029 (0.957) | **1 (0)** |  | -0.6 (0.208) | -0.429 (0.397) | -0.257 (0.623) | 0.086 (0.872) | 0.429 (0.397) | -0.463 (0.355) | NA |
|  | SRL | **-0.829 (0.042)** | -0.657 (0.156) | 0.143 (0.787) | 0.6 (0.208) | -0.314 (0.544) | **-1 (0)** | 0.029 (0.957) | 0.029 (0.957) |  | **0.829 (0.042)** | -0.257 (0.623) | 0.543 (0.266) | **-0.829 (0.042)** | **0.833 (0.039)** | NA |
|  | LCC | **-0.886 (0.019)** | -0.543 (0.266) | 0.143 (0.787) | -0.029 (0.957) | -0.314 (0.544) | -0.657 (0.156) | 0.657 (0.156) | 0.657 (0.156) | 0.657 (0.156) |  | -0.486 (0.329) | 0.714 (0.111) | -0.657 (0.156) | **0.833 (0.039)** | NA |
|  | LNC | -0.371 (0.468) | -0.029 (0.957) | -0.486 (0.329) | 0.771 (0.072) | -0.371 (0.468) | -0.371 (0.468) | -0.2 (0.704) | -0.2 (0.704) | 0.371 (0.468) | 0.029 (0.957) |  | **-0.943 (0.005)** | 0.543 (0.266) | -0.679 (0.138) | NA |
|  | C:N ratio | 0.371 (0.468) | 0.029 (0.957) | 0.486 (0.329) | -0.771 (0.072) | 0.371 (0.468) | 0.371 (0.468) | 0.2 (0.704) | 0.2 (0.704) | -0.371 (0.468) | -0.029 (0.957) | **-1 (0)** |  | -0.714 (0.111) | **0.833 (0.039)** | NA |
|  | Leaf 15N | 0.714 (0.111) | 0.371 (0.468) | 0.257 (0.623) | -0.429 (0.397) | 0.6 (0.208) | 0.6 (0.208) | -0.143 (0.787) | -0.143 (0.787) | -0.6 (0.208) | -0.6 (0.208) | -0.771 (0.072) | 0.771 (0.072) |  | **-0.926 (0.008)** | NA |
|  | Ramets | 0.131 (0.805) | -0.655 (0.158) | 0.131 (0.805) | 0.131 (0.805) | -0.393 (0.441) | -0.393 (0.441) | -0.655 (0.158) | -0.655 (0.158) | 0.393 (0.441) | -0.131 (0.805) | -0.131 (0.805) | 0.131 (0.805) | 0.131 (0.805) |  | NA |
|  | PFI | 0.655 (0.158) | 0.655 (0.158) | 0.655 (0.158) | -0.655 (0.158) | 0.655 (0.158) | 0.655 (0.158) | -0.131 (0.805) | -0.131 (0.805) | -0.655 (0.158) | -0.393 (0.441) | -0.655 (0.158) | 0.655 (0.158) | 0.655 (0.158) | -0.2 (0.704) |  |
